# The ubiquitin ligase E6AP facilitates HDAC6-mediated deacetylation and degradation of tumor suppressors

**DOI:** 10.1038/s41392-020-00330-4

**Published:** 2020-10-19

**Authors:** Yanan Zhang, Zhida Chen, Jing Lin, Jie Liu, Yahong Lin, Huayue Li, Yongyi Xi, Bo Wei, Lihua Ding, Qinong Ye

**Affiliations:** 1 Department of Medical Molecular Biology, Beijing Institute of Biotechnology, Collaborative Innovation Center for Cancer Medicine, Beijing, 100850 PR China; 2 Department of Surgery, The first Medical Center of PLA General Hospital, Beijing, 100853 PR China; 3 Department of Clinical Laboratory, The Fourth Medical Center of PLA General Hospital, Beijing, 100037 PR China

**Keywords:** Oncogenes, Molecular medicine, Oncogenes, Molecular medicine

**Dear Editor,**

Protein acetylation status can regulate protein stability via the ubiquitin-proteasome pathway, which plays a critical role in the regulation of various cellular functions and becomes a target for cancer therapy.^1,2^ Histone deacetylase 6 (HDAC6) belongs to the class II of the histone deacetylase/acuc/apha family and regulates cancer cell proliferation, invasion, and metastasis.^3^ Combination of HDAC6 inhibitors (HDAC6i) with proteasome inhibitors (PI) shows synergistic anticancer activity.^4^ Although many studies reveal how acetyltransferases/deacetylases regulate protein acetylation and subsequent protein ubiquitination and degradation, whether an E3 ubiquitin ligase regulates protein acetylation remains largely unknown. In addition, the mechanisms undelying synergistic anticancer activity of HDAC6i and PI remain poorly understood.

Four and a half LIM domains 1 (FHL1) is a tumor suppressor downregulated in many cancers. Using FHL1 as a bait in the yeast two-hybrid system, we identified HDAC6 and E6-associated protein (E6AP)/UBE3A, an E3 ubiquitin ligase,^5^ as FHL1-interacting proteins (Supplementary Table S1 and Fig. S1a). We further confirmed the interaction among FHL1, HDAC6, and E6AP in mammalian cells and in vitro using different methods (Fig. 1a, b and Supplementary Fig. S1b–g). FHL1, HDAC6, and E6AP formed a complex (Supplementary Fig. S1h). We mapped the regions of the interactions among FHL1, HDAC6, and E6AP (Supplementary Fig. S2a–f).

**Fig. 1:**
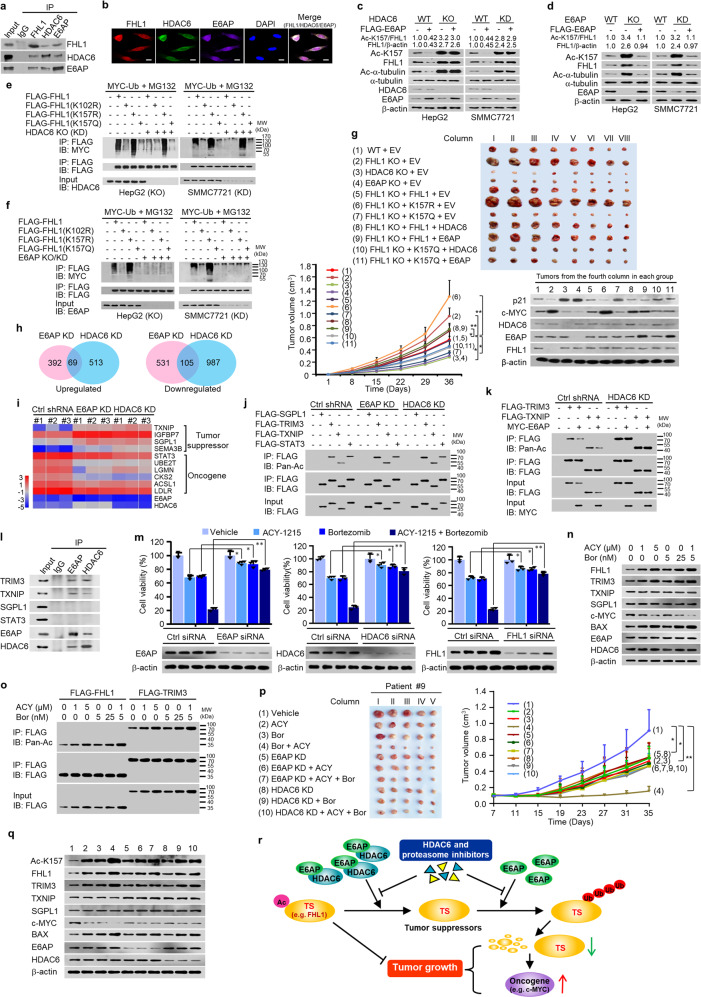
E6AP promotes HDAC6-mediated deacetylation and degradation of tumor suppressors. **a** Interaction among HDAC6, E6AP, and FHL1. Endogenous co-immunoprecipitation (co-IP) was performed with the indicated antibodies or preimmune control serum (IgG) in HepG2 cells, followed by immunoblot. **b** Representative confocal images of HepG2 cells triple labeled for FHL1 (red), HDAC6 (green), and E6AP (purple). The nuclei were stained with DAPI (blue). Scale bar, 10 μm. **c** Immunoblot analysis of HDAC6 wild-type (WT) or knockout (KO) HepG2 cells and HDAC6 WT or knockdown (KD) SMMC7721 cells transfected with empty vector or FLAG-E6AP as indicated. Ac-K157, acetylation at FHL1 K157. The ratio of Ac-K157 to total FHL1 or that of FHL1 to β-actin is shown. β-actin was used as a loading control. **d** Immunoblot analysis of E6AP WT or KO HepG2 cells and E6AP WT or KD SMMC7721 cells transfected with empty vector or FLAG-E6AP as indicated. **e** Ubiquitination analysis of HDAC6 WT or KO HepG2 cells and HDAC6 WT or KD SMMC7721 cells co-transfected with MYC-Ub and FLAG-FHL1 or its mutants and treated with 10 μM MG-132 for 4 h. Cell lysates were immunoprecipitated with anti-FLAG, followed by immunoblot (IB) with anti-MYC. Ub, ubiquitin. **f** Ubiquitination analysis of E6AP WT or KO HepG2 cells and E6AP WT or KD SMMC7721 cells co-transfected with MYC-Ub and FLAG-FHL1 or its mutants and treated with MG-132. Cell lysates were analyzed as in (**e**). **g** Xenograft tumors were established using FHL1 KO HepG2 cells stably infected with lentivirus carrying WT FHL1, FHL1 (K157R) or FHL1 (K157Q) and HDAC6 or E6AP. Stable clones for FHL1, FHL1 (K157R), and FHL1 (K157Q) with similar FHL1 expression levels were used. At the indicated times, the tumors were measured (mean ± SD; *n* = 8), and the growth curve was plotted. Representative immunoblot indicates the expression of c-MYC, p21, FHL1, HDAC6, and E6AP. *P < 0.05, **P < 0.01 at day 36. **h** Proteomic analysis of E6AP and HDAC6 KD HepG2 cells. Overlay plots of upregulated or downregulated protein expression upon E6AP and HDAC6 KD are shown. **i** Heatmap of some sign**i**ficantly regulated proteins by E6AP and HDAC6 as described in (**h**). Ctrl shRNA, control shRNA. **j** Co-IP analysis of WT or E6AP/HDAC6 KD HepG2 cells transfected with the indicated vectors. Pan-Ac, pan-acetyl lysine. E6AP/HDAC6 KD increased acetylation of TRIM3 and TXNIP, but not STAT3. SGPL1 was not acetylated. **k** Co-IP analysis of WT or HDAC6 KD HepG2 cells transfected with the indicated plasmids. **l** Interaction of E6AP and HDAC6 with TRIM3 and TXNIP. Co-IP was performed in HepG2 cells with the indicated antibodies or IgG, followed by immunoblot. **m** Cell viability analysis of HepG2 cells transfected with control siRNA (Ctrl siRNA), E6AP siRNA, HDAC6 siRNA, or FHL1 siRNA and treated with the HDAC6 inhibitor ACY-1215 (1 μM), the proteasome inhibitor bortezomib (5 nM) or ACY-1215 (1 μM) plus bortezomib (5 nM) for 48 h. The knockdown effects were assessed by immunoblot. Data shown are mean ± SD of triplicate measurements that have been repeated three times with similar results. The *P* values were generated using two-way ANOVA. *P < 0.05, **P < 0.01. **n** Immunoblot analysis of HepG2 cells treated with the indicated concentrations of ACY-1215, bortezomib, or ACY-1215 plus bortezomib for 24 h. Bortezomib reduced expression of c-MYC oncogene, which is a known FHL1 and TRIM3 downstream target and is inhibited by FHL1 and TRIM3. **o** Co-IP analysis of HepG2 cells transfected with FLAG-tagged FHL1 or TRIM3 and treated with the indicated concentrations of ACY-1215, bortezomib, or ACY-1215 plus bortezomib for 24 h. **p** The growth curve of liver cancer PDX tumors (Patient #9) treated with ACY-1215, bortezomib, or ACY-1215 plus bortezomib as indicated on lentivirus-mediated shRNA knockdown of E6AP or HDAC6. Data are shown as mean ± SD (*n* = 5). *P < 0.05, **P < 0.01. **q** Immunoblot analysis of representative tumor tissues (the third column) from (**p**). **r** Proposed model for E6AP/HDAC6 modulation of cancer cell sensitivity to HDAC6 and proteasome inhibitors. E6AP and HDAC6, upregulated in various cancers, form a complex and deacetylate tumor suppressors (TS), such as FHL1, leading to the ubiquitination and degradation of the tumor suppressors by E6AP. The decreased tumor suppressors caused increased expression of oncogenes, such as c-MYC, thereby promoting tumor growth. Acetylation of the tumor suppressors (e.g. FHL1) determines their tumor-suppressive function. HDAC6 and proteasome inhibitors block the ability of the E6AP–HDAC6 complex to deacetylate the tumor suppressors, causing decreased ubiquitination and degradation of the tumor suppressors and reduced oncogene expression, and thereby suppress tumor growth

Next, we investigated whether the HDAC6–E6AP complex deacetylates FHL1. HDAC6 knockout (KO) or knockdown (KD) or a HDAC6 inhibitor increased FHL1 acetylation at lysine 157 (Ac-K157), α-tubulin acetylation at lysine 40 (Ac-K40, a known HDAC6 substrate), and FHL1 expression in HepG2 and SMMC7721 liver cancer cells (Supplementary Figs. S3 and S4a, b). HDAC6 overexpression or purified HDAC6 reduced Ac-K157, Ac-K40, and FHL1 expression (Supplementary Fig. S4c–e). Like HDAC6 overexpression, E6AP overexpression decreased Ac-K157, Ac-K40, and FHL1 expression (Fig. 1c and Supplementary Fig. S5a). HDAC6 KO or KD completely abolished the ability of E6AP to decrease Ac-K157, Ac-K40, and FHL1 expression, indicating that E6AP-mediated protein deacetylation depends on HDAC6. Moreover, E6AP KO or KD enhanced Ac-K157, Ac-K40, and FHL1 expression (Fig. 1d and Supplementary Fig. S5b–d). E6AP re-expression in the KO or KD cells rescued this effect (Fig. 1d). E6AP promoted HDAC6-mediated protein deacetylation independently of its E3 ligase activity (C820) (Supplementary Fig. S5e–i). The F785 site of E6AP was critical for the enhancement of HDAC6-mediated deacetylation. Furthermore, E6AP enhances HDAC6-mediated protein deacetylation through increased enzyme–substrate interaction (Supplementary Fig. S6a–f).

Since protein deacetylation can modulate E3 ubiquitin ligase-mediated protein ubiquitination and degradation, we tested how E6AP/HDAC6 decrease FHL1 expression. The results showed that Ac-K157 was critical for FHL1 ubiquitination and degradation, and K102 was the ubiquitination site of FHL1 (Supplementary Fig. S7a–f). In HDAC6/E6AP wild-type (WT) cells, but not in HDAC6/E6AP KO or KD cells, FHL1 (K157R) promoted its ubiquitination, whereas FHL1 (K157Q) reduced its ubiquitination (Fig. 1e, f). HDAC6/E6AP KO or KD inhibited FHL1 ubiquitination but had no effect on the ubiquitination of FHL1 (K102R), FHL1 (K157R), or FHL1 (K157Q) except for FHL1 (K157R) on E6AP KO. This is due to the lack of ubiquitination or acetylation site in these mutants or lack of the E3 ligase activity. Similar trends were observed with endogenous FHL1 ubiquitination (Supplementary Fig. S7g, h). E6AP did not affect HDAC6 ubiquitination and expression (Supplementary Fig. S7i). Mechanistically, FHL1 deacetylation promoted its ubiquitination through enhanced E6AP–FHL1 interaction (Supplementary Fig. S8a–g). Taken together, these data suggest that FHL1 deacetylation at K157 is critical for its K102 ubiquitination via E6AP.

FHL1 was shown to inhibit hepatocellular carcinoma (HCC) growth via inhibition of oncogene c-MYC and activation of tumor suppressor p21. Interestingly, Ac-K157 was critical for FHL1 anti-proliferation activity (Supplementary Fig. S9a, b and Fig. 1g). HDAC6/E6AP enhanced cancer cell proliferation in vitro and in nude mice largely through FHL1 deacetylation at K157 (Supplementary Fig. S9c, d and Fig. 1g). In HCC patients, E6AP and HDAC6 were upregulated, and FHL1 and Ac-K157 were downregulated (Supplementary Figs. S10 and S11a, b). E6AP and HDAC6 expression negatively correlated with FHL1 and Ac-K157 expression, and E6AP positively correlated with HDAC6 (Supplementary Fig. S11c). HCC patients with high E6AP or HDAC6 expression had shorter disease-free survival (DFS) and overall survival (OS), and those with high FHL1 or Ac-K157 expression had longer DFS and OS (Supplementary Fig. S11d).

To test if protein deacetylation by the E6AP–HDAC6 complex is a general mechanism, we explored additional E6AP/HDAC6 substrates. Yeast two-hybrid screen identified the tumor suppressors TRIM3 and TXNIP as binding partners of both E6AP and HDAC6 (Supplementary Fig. S12a and Tables S2, S3). Proteomic analysis combined with immunoblot showed that tumor suppressors TXNIP and SGPL1 might be potential E6AP and HDAC6 targets (Fig. 1h, i and Supplementary Fig. S12b–e). Further analyses revealed that E6AP decreased TRIM3 and TXNIP acetylation in HDAC6-dependent and E3 ligase activity-independent manners (Fig. 1j, k and Supplementary Fig. S12f–j). E6AP and HDAC6 interacted with TRIM3 and TXNIP, and formed complexes with TRIM3 and TXNIP, respectively (Fig. 1l and Supplementary Fig. S12k–m). Like FHL1 knockdown, TRIM3 knockdown promoted HCC cell proliferation, with increased c-MYC expression and decreased p21 expression (Supplementary Fig. S12n).

Next, we examined if E6AP and HDAC6 together with their common substrates affect cancer cell sensitivity to HDAC6i and PI. KD of E6AP, HDAC6, FHL1, or TRIM3, but not TXNIP, SGPL1, HDAC10, or p53 (a well-known tumor suppressor), decreased the sensitivity of HCC cells to HDAC6i or PI (Supplementary Fig. S13a–d). Both E6AP and HDAC6 expression negatively correlated with half maximal inhibitory concentration (IC_50_) of the HDAC6 inhibitor ACY1215 and the PI bortezomib in 13 liver cancer cell lines (Supplementary Fig. S13e). KD of E6AP, HDAC6, or FHL1 also attenuated the synergistic effect of ACY1215 and bortezomib on viability of HCC cells (Fig. 1m and Supplementary Fig. S13f). Intriguingly, ACY1215 and bortezomib increased expression of FHL1, TRIM3, and Bax, a previously reported E6AP/HDAC6 target (Fig. 1n and Supplementary Fig. S13g). Moreover, the synergistic effect of ACY1215 and bortezomib on expression and acetylation of FHL1 and TRIM3 was observed (Fig. 1n, o and Supplementary Fig. S13g, h). Similar results were observed in patient-derived xenograft (PDX) mouse models and cell line-derived xenograft mouse models (Fig. 1p, q and Supplementary Fig. S14a–d, and Table S4). In PDX models, patients with high E6AP and HDAC6 expression generally benefited more from the treatment of ACY1215 and bortezomib, alone or combination, than those with low E6AP and HDAC6 expression.

In summary, we show that the E3 ubiquitin ligase E6AP promotes HDAC6-mediated deacetylation of tumor suppressors, such as FHL1, TRIM3, and TXNIP, in an E3 ligase activity-independent manner, causing decreased expression of these tumor suppressors. However, which factors (e.g. hepatitis B virus infection) drive HDAC6–E6AP complex formation and their activities remain to be investigated. FHL1 deacetylation by the E6AP/HDAC6 heterodimer abolishes its tumor-suppressive function. Inhibitors of HDAC6 and proteasome synergistically increase acetylation and expression of the tumor suppressors suppressed by E6AP/HDAC6 (Fig. 1r). These findings suggest a role for an E3 ligase in the regulation of protein acetylation and imply that E6AP–HDAC6 complex may be an attractive target for cancer therapy.

## Supplementary information

Supplemental material

## Data Availability

Data are available upon reasonable request.
