# Supplementary material for: The ubiquitin ligase E6AP facilitates HDAC6-mediated deacetylation and degradation of tumor suppressors
Source: Signal Transduct Target Ther. 2020 Oct 19;5:243. doi: 10.1038/s41392-020-00330-4 (PMC7573597; doi:10.1038/s41392-020-00330-4)
Supplement: Supplementary file 1 — Supplemental material [file 41392_2020_330_MOESM1_ESM.docx]

Supplementary Materials for

The ubiquitin ligase E6AP facilitates HDAC6-mediated deacetylation and degradation of tumor suppressors

Yanan Zhang^1#^, Zhida Chen^1,2#^, Jing Lin^3#^, Jie Liu^1#^, Yahong Lin^1^, Huayue Li^1^, Yongyi Xi^1^, Bo Wei^1,2*^, Lihua Ding^1*^ and Qinong Ye^1*^

Correspondence to: Qinong Ye (yeqn66@yahoo.com) or Lihua Ding (dinglh@126.com) or Bo Wei (weibo@vip.163.com)

**This PDF file includes:**

Materials and Methods

Figures. S1 to S14

Tables S1 to S5

Materials and Methods

**Plasmids, siRNAs, shRNAs, Lentiviruses and reagents**

The eukaryotic expression vectors with FLAG, MYC or HA epitope were constructed by inserting PCR-amplified gene fragments into pcDNA3 (Invitrogen) with FLAG, MYC or HA tag, respectively. Prokaryotic plasmids encoding GST or His fusion proteins were generated by cloning PCR-amplified sequences into pGEX-KG (Amersham Pharmacia Biotech) or pET-28a (Novagen), respectively. The mutants for the FLAG-, MYC- or GST-tagged proteins were made by recombinant PCR. Lentiviral vectors for FHL1, FHL1 (K157Q), FHL1 (K157R), E6AP and HDAC6 were obtained by inserting PCR-amplified gene fragments into pCDH (System Biosciences). The cDNA target sequences of siRNAs and/or shRNAs for E6AP, UBE3B, UBE3C, HDAC6, HDAC10, FHL1, TRIM3, TXNIP, and SGPL1 were listed in Supplmentary Table S5. Lentiviral vectors for gene overexpression were constructed by inserting PCR-amplified gene fragments into pCDH (System Biosciences). Lentiviral shRNA vectors were made by cloning short hairpin RNA fragments into pSIH-H1-Puro (System Biosciences). Lentiviruses were produced by co-transfecting HEK293T cells with recombinant lentiviral vectors and pPACK Packaging Plasmid Mix (System Biosciences) using Megatran reagent (Origene) according to the manufacture’s protocols. Viral supernatants were harvested 48 h after transfection and the titer was detected. The target cells were then infected with the lentiviral constructs with 8 μg/ml polybrene (Sigma-Aldrich). Stable cell lines were selected in 1 μg/ml puromycin for approximately one month, and E6AP and HDAC6 knockdown cell lines were generated.

The Ac-K157 antibody was made by Abmart. The designed peptide of FHL1, EDFYCVTCHETK(Ac)FAKHCVKCNKAI, was used as an antigen to raise polyclonal antibodies in rabbit. Anti-c-MYC (sc-764), anti-E6AP (sc-25509), anti-HDAC6 (sc-28386), anti-FHL1 (sc-133580), anti-p21 (sc-397), anti-Ubiquitin (sc-8017), anti-β-actin (sc-47778HRP), anti-UBE2T (sc-100623), anti-PML (sc-5621), anti-MSH2 (sc-22771) and anti-BAX (sc-7480) were purchased from Santa Cruz Biotechnology. Anti-FLAG (A8592), anti-FLAG M2 agarose (A2220), anti-c-MYC gel (E6654) and anti-c-MYC-peroxidase (A5598) were from Sigma-Aldrich. Anti-GST (RPN1236) and anti-His (27-4710-01) were obtained from GE Healthcare. Anti-FHL1 (10991-1-AP), anti-HDAC6 (12834-1-AP), anti-E6AP (60038-1-Ig) and anti-α-tubulin (11224-1-AP) were from Proteintech. Anti-acetyl α-tubulin K40 (5335), anti-Pan-acetylated-lysine (9441) and anti-CDK1 (77055) were purchased from Cell Signaling. Anti-TRIM3 (GTX106410), anti-TXNIP (GTX45803) and anti-IGFBP7 (GTX88132) were from GeneTex.

**Human clinical samples**

One hundred and twenty-eight primary liver carcinomas and adjacent noncancerous tissues used for examination of clinical relevance of HDAC6/E6AP-mediated FHL1 deacetylation axis and 15 primary liver carcinomas used for the generation of PDX mouse models were collected from Chinese PLA General Hospital, with the informed consent of patients and with the approval of the Institutional Review Committees of Chinese PLA General Hospital. Similar experiments performed previously were used to estimate sample size. All cases used for the clinical relevance study include 102 males and 26 females with 34-66 years of age (mean age: 51.3 years). The follow-up time was 5.7-68.3 months (mean: 30.0 months). Normal distribution was performed using SPSS13.0. All patients used for the generation of PDX models include 12 males and 3 females with 38-66 years of age (mean age: 54.6 years) (Supplementary Table S4).

**Mice**

Animal studies follow an animal use protocol approved by the Institutional Animal Care and Use Committee of Beijing Institute of Biotechnology. For cell line-derived xenograft models, ten million liver cancer cells were subcutaneously injected into the hind flanks of six-week-old male BABL/c nude mice. For the generation of PDX models, fresh liver tumor tissue specimens were rinsed twice with PBS containing antibiotic (100 U/ml penicillin and 0.1 mg/ml streptomycin) and cut into 3 × 3 × 3 mm pieces, and then implanted subcutaneously into the flanks of six-week-old NOD/SCID mice. Successfully engrafted tumors were confirmed with immunohistology analysis, and banked after two passages.

For *in vivo* drug treatment, mice were allotted randomly into different groups when tumors reached around 100 mm^3^. The mice were intraperitoneally (i.p.) injected with ACY-1215 (50 mg/kg), Bortezomib (0.5 mg/kg) or ACY-1215 (50 mg/kg) plus Bortezomib (0.5 mg/kg) every 3 days for 10 times. Controls received vehicle. To determine the effect of E6AP and HDAC6 expression on sensitivity of liver cancer cells to ACY-1215 and Bortezomib treatment, the PDX mice were intratumorally injected with 50 μl of lentiviruses harboring control shRNA, E6AP shRNA or HDAC6 shRNA (4 × 10^7^ TU/ml) every 3 days for 10 times as indicated. Tumor growth was monitored at the indicated time points. Tumor volume was calculated according to the following formula: volume = (longest diameter × shortest diameter^2^)/2. Excised tumors were weighed, and portions were frozen in liquid nitrogen or fixed in 4% paraformaldehyde for further study. Similar experiments done previously were used to estimate sample size.

**FHL1, E6AP and HDAC6 knockout cancer cell lines**

FHL1, E6AP and HDAC6 knockout cancer cells were generated by CRISPR/Cas9 (Genloci Biotechnologies Inc.). CRISPRs were designed using a CRISPR design web tool (http://crispr.mit.edu). The sgRNA (single guide RNA) sequences targeted by FHL1, E6AP and HDAC6 CRISPR are GTGTGGAATGCCGCAAGCCC, CAACCGGCCAGGATTCCACC and AAACAAGAAAGGTCCTCGAG, respectively. The sgRNAs were cloned into the lentiCRISPRv2 vector (addgene #52961). The recombinant lentiviruses were produced by co-transfecting HEK293T cells using Megatran reagent (Origene). The target cells were infected with the lentiviral constructs with 8 μg/ml polybrene (Sigma-Aldrich). Stable cell lines were selected with 1 μg/ml puromycin. Cells were screened for mutations at nuclease target sites by PCR amplification of genomic sequences, followed by DNA sequencing and immunoblot.

**E6AP^+/+^ and E6AP^-/-^ mouse embryonic fibroblasts**

Male E6AP^+/-^ mouse (Jackson Laboratory; strain 129-Ube3atm1Alb/J) was mated naturally with female E6AP^+/-^ mouse to yield E6AP^-/-^ mouse. E6AP^-/-^ fetal mice were harvested at day E13–E14 of pregnancy. Mouse embryonic fibroblasts (MEFs) were isolated as previously described.^1^ Briefly, tissues from the fetal mice were incubated in trypsin solution to obtain a single-cell suspension. The suspension was then washed twice in MEF medium and incubated at 37°C.

**Cell lines and drug treatments**

Human embryonic kidney HEK293T cells, human liver cancer HepG2, SMMC7721, MHCC-97H, Hep3B, BEL-7402, SNU-387, SNU-182, SK-HEP-1, Li-7, Huh-7, and PLC/PRF/5 cells, and mouse hepatocarcinoma HEPA1-6 cells were purchased from American Type Culture Collection (ATCC), and have previously been examined for mycoplasma contamination. Cells were routinely maintained in DMEM or RPMI-1640 (Invitrogen) containing 10% FBS (Hyclone). Lipofectamine 3000 reagent (Invitrogen) and Lipofectamine RNAiMAX (Invitrogen) were used for transfections of plasmids and siRNAs, respectively. For transfection of plasmids only, cells were collected 24 h after transfection for further study. For siRNA transfection, cells were harvested at 48 h post-transfection for further study. For transfection of siRNAs plus plasmids, cells were first transfected with siRNAs. Twenty-four hours later, the cells were then transfected with plasmids and collected 24 h after transfection. For drug treatment, transfected cells were re-plated into 96-well plates at 3000 cells/well, and treated with indicated concentrations of ACY-1215, ACY-738, Tubacin, FK228, Bortezomib, Ixazomib, or carfilzomib, or a combination. Forty-eight hours after treatment, cell viability was examined by the CCK-8 Kit (Dojindo Laboratories) according to the manufacturer’s protocols. Briefly, ten microliters of CCK-8 solution were added to cultured cells in each well, followed by incubation at 37°C for 1 h. The OD values were measured at 450 nm using a microplate reader. The half maximal inhibitory concentration (IC50) of a drug was calculated using the Graphpad prism 7.0.

**Yeast two-hybrid system**

The bait plasmid pAS2-FHL1, pAS2-E6AP or pAS2-HDAC6 and a human mammary two-hybrid cDNA library (Clontech) were sequentially transformed into Saccharomyces cerevisiae strain CG1945 according to the manufacturer’s protocols (Clontech). Transformants were cultured on synthetic medium lacking tryptophan, leucine and histidine but containing 1mM 3-aminotriazole. The candidate clones were isolated from the yeast cells and re-transformed back to the same yeast strain to confirm the interaction of the candidates with the bait. The specificity of the interaction was examined by comparing the associations between the candidates and various bait constructs. The unrelated bait construct pAS2-lamin C was used as a negative control.

**Co-immunoprecipitation and GST pull-down assays**

For co-immunoprecipitation (co-IP) assay, cells were harvested, rinsed in PBS, and lysed in lysis buffer (50 mM Tris at pH 8.0, 500 mM NaCl, 0.5% Nonidet P-40, 1 mM dithiothreitol and protease inhibitors). The protein extracts were then immunoprecipitated with antibody or control serum (Santa Cruz Biotechnology) according to the manufacturer’s instructions. The precipitated proteins were separated and detected with standard immunoblot. For GST pull-down assay, GST or His fusion proteins were expressed and purified according to the manufacturers’ protocols (Amersham Pharmacia and Qiagen). Expression of His and GST fusion proteins in E. coli BL21 (DE3) was induced by 0.1 mM isopropyl β-D-thiogalactoside (IPTG) for 20 h at 20 °C. His or GST fusion proteins were purified with nickel beads (Qiagen) or glutamine Sepharose 4B beads (Amersham Pharmacia), respectively. The bound His fusion proteins were eluted with His elution buffer (50 mM NaH2PO4, 300 mM NaCl, 500 mM imidazole pH 8.0). Recombinant human FHL1 protein without GST tag was obtained from glutamine Sepharose 4B beads containing purified GST-FHL1 cleaved by thrombin (GE Healthcare) at 24 °C overnight in 1 × cleavage buffer (20 mM Tris-HCl pH 8.4, 150 mM NaCl, 2.5 mM CaCl2, 1 mM DTT). The eluted His fusion proteins and recombinant human FHL1 protein were dialyzed using binding buffer (50mM Tris-HCl pH 7.5, 150 mM NaCl, 1 mM EDTA, 0.3 mM dithiothreitol, 0.1% Nonidet P-40 and protease inhibitors) at 4 °C overnight. Purified His fusion proteins, recombinant human FHL1 protein or cell lysates were incubated with GST fusion protein bound to glutamine Sepharose 4B beads for 4 h under rotation at 4°C in 500 μl of binding buffer. The beads were precipitated, washed four times with binding buffer, eluted by boiling in SDS sample buffer and analyzed by immunoblot analysis.

**Dot blot**

Grids were drawn on a nitrocellulose membrane by a pencil to indicate dots. Samples were spotted onto the nitrocellulose membrane at the center of the grid, which was allowed to dry for 1 h at the room temperature. The membrane was rinsed briefly in PBS, and was incubated overnight at 4°C in 5% BSA in [TBST 20 mM Tris-Cl pH7.6, 150 mM NaCl, 0.05%（V/V）Tween 20] to block non-specific binding sites on the membrane. The membrane was incubated with primary antibody dissolved in TBST for 30 min at room temperature, followed by incubation with secondary antibody conjugated with HRP for 30 min at room temperature. After washing, the chemiluminescence detection system was used to obtain the signals.

***In vitro* deacetylation assay**

GST or His fusion proteins were expressed and purified according to the manufacturers’ instructions (Amersham Pharmacia and Qiagen) as described above. The bound His fusion proteins were eluted with His elution buffer (50 mM NaH_2_PO4, 300 mM NaCl, 500 mM imidazole pH8.0) and the bound GST fusion proteins were eluted with GST elution buffer (50 mM Tris-HCl pH 8.7, 150 mM NaCl, 50 mM reduced glutathione, 0.5 mM DTT, 0.1% CHAPS, pH 8.0). The eluted proteins were dialyzed using deacetylation buffer (40 mM HEPES pH 7.0, 6 mM MgCl_2_, 1 mM DTT) overnight for further experiments. To perform the in vitro deacetylation assay, 0.25 μg of acetylated or unacetylated FHL1 or α-tubulin peptides were incubated with indicated amounts of recombinant HDAC6 or E6AP or HDAC6 plus E6AP in the deacetylation buffer at 37 °C for 1 h. The reaction mixtures were spotted on nitrocellulose membrane to detect deacetylation of FHL1 or α-tubulin peptides by dot blot with anti-Ac-K157 for FHL1 or anti-Ac-K40 for α-tubulin.

**Mass spectrometry**

For analysis of FHL1 acetylation, HepG2 cells stably expressing FLAG-FHL1 were lysed and immunoprecipitated using FLAG M2-conjugated affinity gel, followed by elution with FLAG peptide according to the manufacturer’s instructions (Sigma-Aldrich). The eluted material was resolved by SDS–PAGE and visualized by silver staining. The bands were cut from SDS–PAGE gel, fully trypsinized, and analyzed by nano-liquid chromatography-tandem mass spectrometry (nanoLC-MS/MS) (nanoACQUITY UPLC and SYNAPT G2 HD mass spectrometer, Waters). MS/MS data were obtained with Data Dependent Analysis mode and processed with PLGS 2.4 software (Waters), and the resulting peak list was searched against the NCBI database with the MASCOT search engine.

For analysis of proteins altered upon E6AP or HDAC6 KD in HepG2 cells, isobaric tags for relative and absolute quantification (iTRAQ) and label free quantitative proteomic approaches were performed. Briefly, the WT, E6AP KD and HDAC6 KD HepG2 cells were lysed in a buffer containing 8 M Urea. Protein samples (50 μg) in triplicate were digested with trypsin and subsequently identified and quantified using nano-LC-MS/MS and label-free quantification. The LC-MS/MS detection system was composed of a nanoflow high-performance liquid chromatograph (HPLC) instrument (Thermo Scientific EASY-nLC 1000 system) coupled to a Orbitrap Fusion mass spectrometer (Thermo Fisher) with a nanoelectrospray ion source (Thermo Fisher). The liquid chromatography columns were packed using a 120-min linear gradient. The system was operated in the data-dependent mode in which the MS/MS fragmentation was conducted using the 20 most intense peaks from each MS scan. Proteins were identified by MaxQuant-based database searching. The tandem mass spectra were searched against the human protein database (Uniport, human). MS/MS spectra were searched with a maximum allowed fragment ion mass tolerance to ± 20 mmu. Carbamidomethylation of cysteine residues was set as a fixed modification, while oxidation of methionine residues and protein N-terminal acetylation were set as variable modifications. Trypsin cleavage with 2 miscleavages was allowed. The false discovery rate (FDR) was set to 1%. A fold-change of ≥ 2.0 or ≤ 0.5 and P-value < 0.05 in 3 replicates were considered significant. The mass spectrometry proteomics data have been deposited to the ProteomeXchange Consortium (http://proteomecentral.proteomexchange.org) via the iProX partner repository with the dataset identifier PXD017955.^2^

**Ubiquitination assay**

For *in vivo* ubiquitination assay, transfected cells were treated with the proteasome inhibitor MG132 (10 μM) for 4 h. The cells were lysed in lysis buffer and immunoprecipitated with anti-FLAG or anti-FHL1. The immunocomplexes were subjected to immunoblot analysis with indicated antibodies. In vitro ubiquitination assay was carried out using the Human E6AP/E6 Ubiquitin Ligase Kit (R&D) according to the manufacturer’s instructions. Briefly, recombinant FHL1 or its mutant was incubated in a reaction buffer containing E1, E2 (UBCH7), E3 (UBE3A/E6AP) and ubiquitin at 37°C for 2 h. The reaction was terminated by adding the gel loading buffer, followed by immunoblot.

**Immunofluorescence**

Cells grown on glass coverslips were fixed and permeabilized. Endogenous peroxidase activity was quenched by treatment with 3% H_2_O_2_ for 15 min. Sections were blocked with normal serum for 30 min. The coverslips were then incubated with mouse anti-HDAC6 (sc-28386, Santa Cruz Biotechnology), mouse anti-E6AP (60038-1-Ig, Proteintech), rabbit anti-FHL1 (10991-1-AP, Proteintech) at 4°C overnight. For triple immunofluorescence, horse raddish peroxidase (HRP)-conjugated secondary antibodies (DS-0001, PV-6001 and PV-9001 or PV-9003, Zhongshan Biotech) were applied at 37°C for 1 h, and then immunoreactive cells were visualized with Fluorescein Amplification Reagent (NEL741001KT, NEL744001KT and NEL745001KT, Perkinelmer). After the first fluorescence precipitated, the primary and secondary antibodies were inactivated in citrate buffer (pH 6.0) with a microwave oven. The second and third fluorescence was then performed as above. Nuclei were counterstained with 4’,6-diamidino-2-phenylindole (DAPI). Confocal images were collected using a Radiance2100 confocal microscope (Bio-Rad).

**Quantitative reverse transcription-PCR (RT-qPCR)**

Total RNA was isolated using TRIzol reagent (Invitrogen) and reverse transcription was performed by Quantscript RT kit (Tiangen) according to the manufacturer’s protocols. Real-time quantitative PCR was performed with SYBR-green dye on a CFX96 Real-Time PCR detection system (Bio-Rad). The primers for FHL1 amplification are 5’-GACTGGAAGCTTCTTCCCTAAAG-3’ and 5’-CCAGCTTCTTAGAGCAGGTAA CA-3’, and the primers for β-actin are 5’-AGACTTCGAGCAGGAGATGG-3’ and 5’-CGGATGTCAACGTCACACTT-3’. The relative quantification value of the target, normalized to β-actin, was calculated by the comparative Ct methods.

**Cell proliferation**

Three thousand cells were seeded per well in 96 well plates in regular cell growth media. Cell proliferation was examined by the CCK-8 Kit (Dojindo Laboratories) according to the manufacturer’s protocols. Briefly, ten microliters of CCK-8 solution were added to cultured cells in each well, followed by incubation at 37 °C for 1 h. The absorbance at 450 nm was determined using a microplate reader, which represents relative cell number.

**Immunohistochemistry**

Immunohistochemistry (IHC) of formalin-fixed paraffin-embedded samples was performed as described previously.^3^ Briefly, the formalin-fixed paraffin sections were deparaffinized, rehydrated, and pretreated with 3% H_2_O_2_ for 20 min. The antibody-binding epitopes of the antigens were retrieved by microwave treatment, and the sections were then preincubated with 10% goat serum to block nonspecific binding. Mouse anti-HDAC6 (sc-28386, Santa Cruz Biotechnology), mouse anti-E6AP (60038-1-Ig, Proteintech), rabbit anti-FHL1 (10991-1-AP, Proteintech), and rabbit anti-Ac-K157 were used at dilutions of 1:100 as the primary antibodies for IHC. FHL1, Ac-K157, HDAC6 or E6AP score was generated by multiplying the percentage of stained cells (0–100%) by the intensity of the staining (low, 1+; medium, 2+; strong, 3+). Thus, the score is between 0–3. The optimal cutoff values of the IHC scores were determined using receiver operating characteristic (ROC) curve analysis. We defined score < 1.5 as low Ac-K157, E6AP and HDAC6 and FHL1 score < 1.0 as low.

**Statistical analysis**

Trial experiments or similar experiments done previously were used to assess sample size with adequate statistical power. Disease-free survival and overall survival curves were generated by the Kaplan-Meier method and differences between survival curves were determined using the log-rank test. Data were analyzed with two-tailed student’s t-test for two comparisons or one-way ANOVA test with Bonferroni correction for multiple comparisons. The correlation between E6AP or HDAC6 expression and the endpoint tumor growth inhibition rate or the IC50 of ACY-1215 or Bortezomib was determined using Pearson’s correlation test. All statistical tests were two-sided. Statistical calculations were performed using GraphPad Prism7.0. In all assays, *P* < 0.05 were considered statistically significant.

**REFERENCES**

1. Okita, K., Hong, H., Takahashi, K. & Yamanaka, S. Generation of mouse-induced pluripotent stem cells with plasmid vectors. *Nat. Protoc.* **5**, 418-428 (2010).

2. Ma, J. et al. iProX: an integrated proteome resource. *Nucleic Acids Res.* **47**, D1211-D1217 (2019).

3. Zhang, H. et al. Stimulatory cross-talk between NFAT3 and estrogen receptor in breast cancer cells. *J. Biol. Chem.* **280**, 43188-43197 (2005).

Figure. S1.


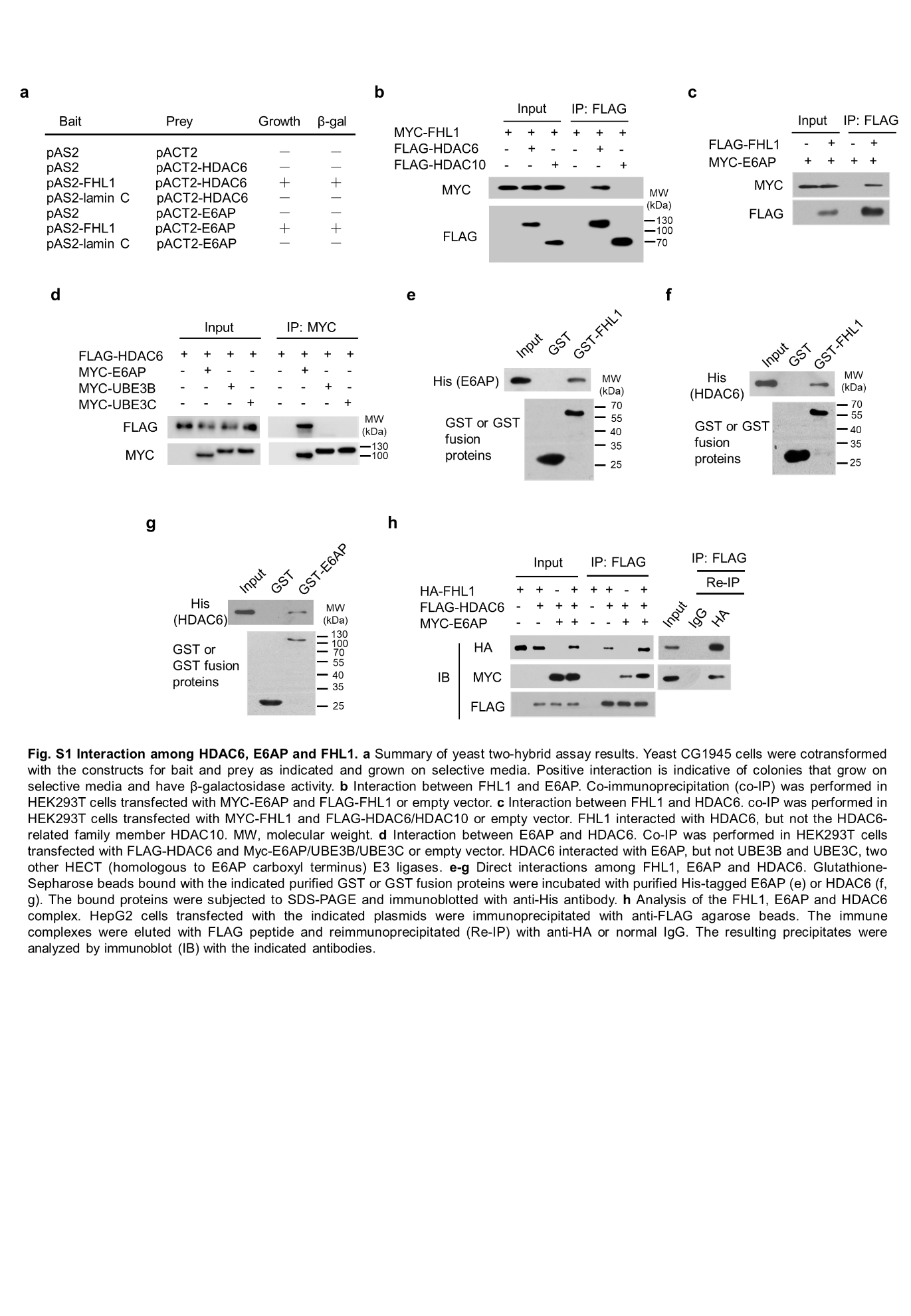


**Figure. S1 Interaction among HDAC6, E6AP and FHL1.** **a** Summary of yeast two-hybrid assay results. Yeast CG1945 cells were cotransformed with the constructs for bait and prey as indicated and grown on selective media. Positive interaction is indicative of colonies that grow on selective media and have β-galactosidase activity. **b** Interaction between FHL1 and E6AP. Co-immunoprecipitation (co-IP) was performed in HEK293T cells transfected with MYC-E6AP and FLAG-FHL1 or empty vector. **c** Interaction between FHL1 and HDAC6. co-IP was performed in HEK293T cells transfected with MYC-FHL1 and FLAG-HDAC6/HDAC10 or empty vector. FHL1 interacted with HDAC6, but not the HDAC6-related family member HDAC10. MW, molecular weight. **d** Interaction between E6AP and HDAC6. Co-IP was performed in HEK293T cells transfected with FLAG-HDAC6 and Myc-E6AP/UBE3B/UBE3C or empty vector. HDAC6 interacted with E6AP, but not UBE3B and UBE3C, two other HECT (homologous to E6AP carboxyl terminus) E3 ligases. **e-g** Direct interactions among FHL1, E6AP and HDAC6. Glutathione-Sepharose beads bound with the indicated purified GST or GST fusion proteins were incubated with purified His-tagged E6AP (e) or HDAC6 (f, g). The bound proteins were subjected to SDS-PAGE and immunoblotted with anti-His antibody. **h** Analysis of the FHL1, E6AP and HDAC6 complex. HepG2 cells transfected with the indicated plasmids were immunoprecipitated with anti-FLAG agarose beads. The immune complexes were eluted with FLAG peptide and reimmunoprecipitated (Re-IP) with anti-HA or normal IgG. The resulting precipitates were analyzed by immunoblot (IB) with the indicated antibodies.

Figure. S2.


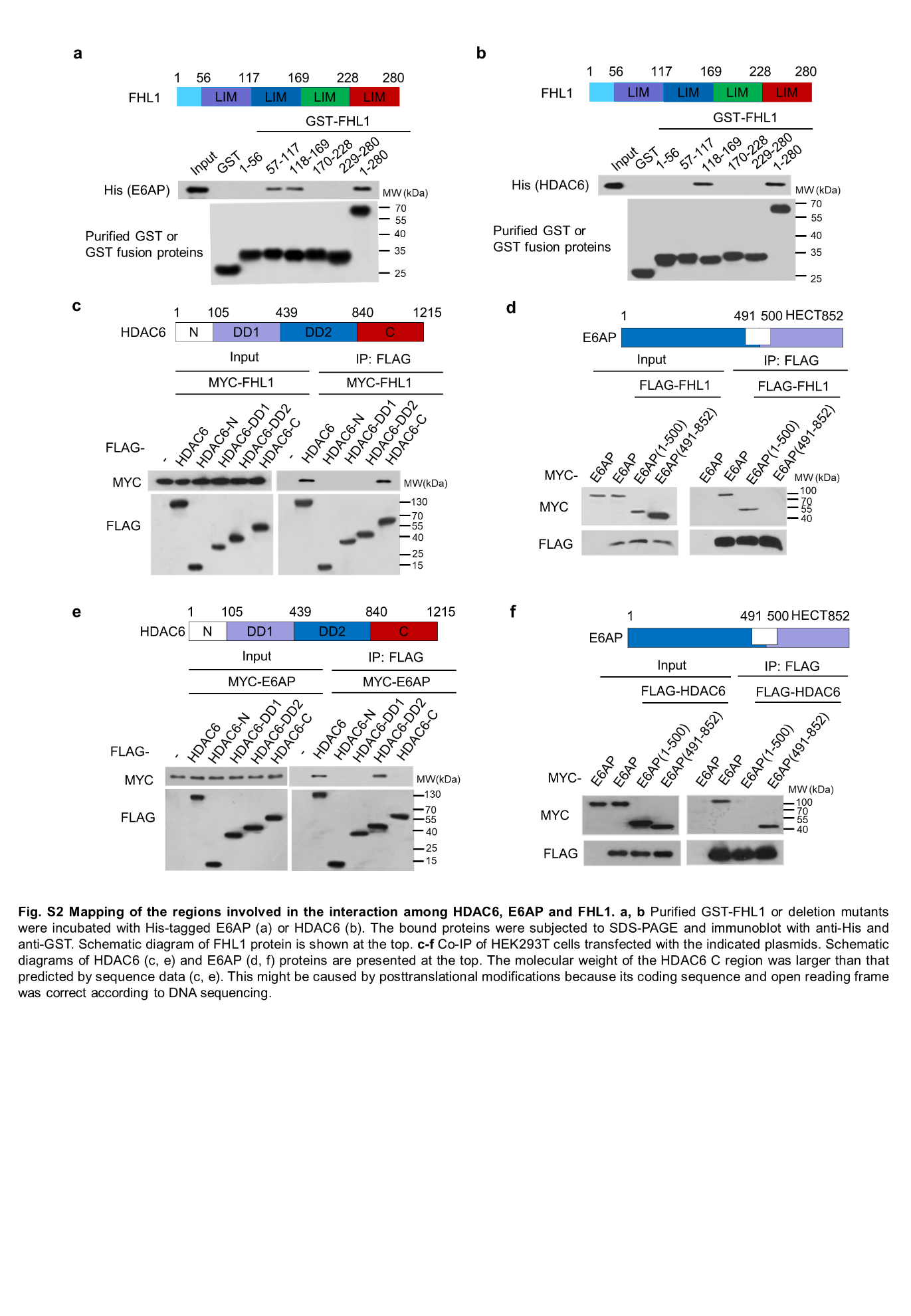


**Figure. S2 Mapping of the regions involved in the interaction among HDAC6, E6AP and FHL1. a, b** Purified GST-FHL1 or deletion mutants were incubated with His-tagged E6AP (a) or HDAC6 (b). The bound proteins were subjected to SDS-PAGE and immunoblot with anti-His and anti-GST. Schematic diagram of FHL1 protein is shown at the top. **c-f** Co-IP of HEK293T cells transfected with the indicated plasmids. Schematic diagrams of HDAC6 (c, e) and E6AP (d, f) proteins are presented at the top. The molecular weight of the HDAC6 C region was larger than that predicted by sequence data (c, e). This might be caused by posttranslational modifications because its coding sequence and open reading frame was correct according to DNA sequencing.

Figure. S3.


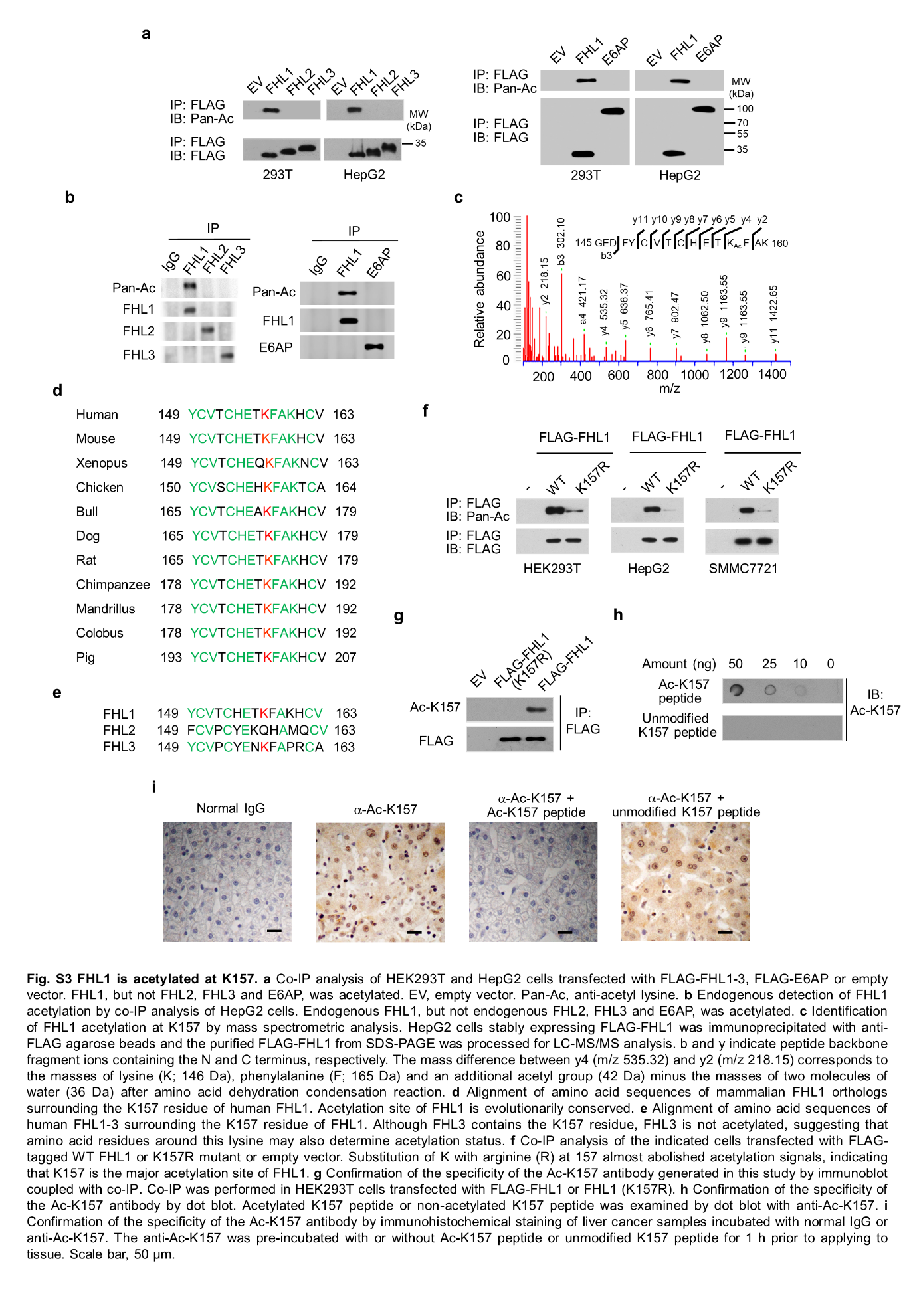


**Figure. S3 FHL1 is acetylated at K157. a** Co-IP analysis of HEK293T and HepG2 cells transfected with FLAG-FHL1-3, FLAG-E6AP or empty vector. FHL1, but not FHL2, FHL3 and E6AP, was acetylated. EV, empty vector. Pan-Ac, anti-acetyl lysine. **b** Endogenous detection of FHL1 acetylation by co-IP analysis of HepG2 cells. Endogenous FHL1, but not endogenous FHL2, FHL3 and E6AP, was acetylated. **c** Identification of FHL1 acetylation at K157 by mass spectrometric analysis. HepG2 cells stably expressing FLAG-FHL1 was immunoprecipitated with anti-FLAG agarose beads and the purified FLAG-FHL1 from SDS-PAGE was processed for LC-MS/MS analysis. b and y indicate peptide backbone fragment ions containing the N and C terminus, respectively. The mass difference between y4 (m/z 535.32) and y2 (m/z 218.15) corresponds to the masses of lysine (K; 146 Da), phenylalanine (F; 165 Da) and an additional acetyl group (42 Da) minus the masses of two molecules of water (36 Da) after amino acid dehydration condensation reaction. **d** Alignment of amino acid sequences of mammalian FHL1 orthologs surrounding the K157 residue of human FHL1. Acetylation site of FHL1 is evolutionarily conserved. **e** Alignment of amino acid sequences of human FHL1-3 surrounding the K157 residue of FHL1. Although FHL3 contains the K157 residue, FHL3 is not acetylated, suggesting that amino acid residues around this lysine may also determine acetylation status. **f** Co-IP analysis of the indicated cells transfected with FLAG-tagged WT FHL1 or K157R mutant or empty vector. Substitution of K with arginine (R) at 157 almost abolished acetylation signals, indicating that K157 is the major acetylation site of FHL1. **g** Confirmation of the specificity of the Ac-K157 antibody generated in this study by immunoblot coupled with co-IP. Co-IP was performed in HEK293T cells transfected with FLAG-FHL1 or FHL1 (K157R). **h** Confirmation of the specificity of the Ac-K157 antibody by dot blot. Acetylated K157 peptide or non-acetylated K157 peptide was examined by dot blot with anti-Ac-K157. **i** Confirmation of the specificity of the Ac-K157 antibody by immunohistochemical staining of liver cancer samples incubated with normal IgG or anti-Ac-K157. The anti-Ac-K157 was pre-incubated with or without Ac-K157 peptide or unmodified K157 peptide for 1 h prior to applying to tissue. Scale bar, 50 μm.

Figure. S4.


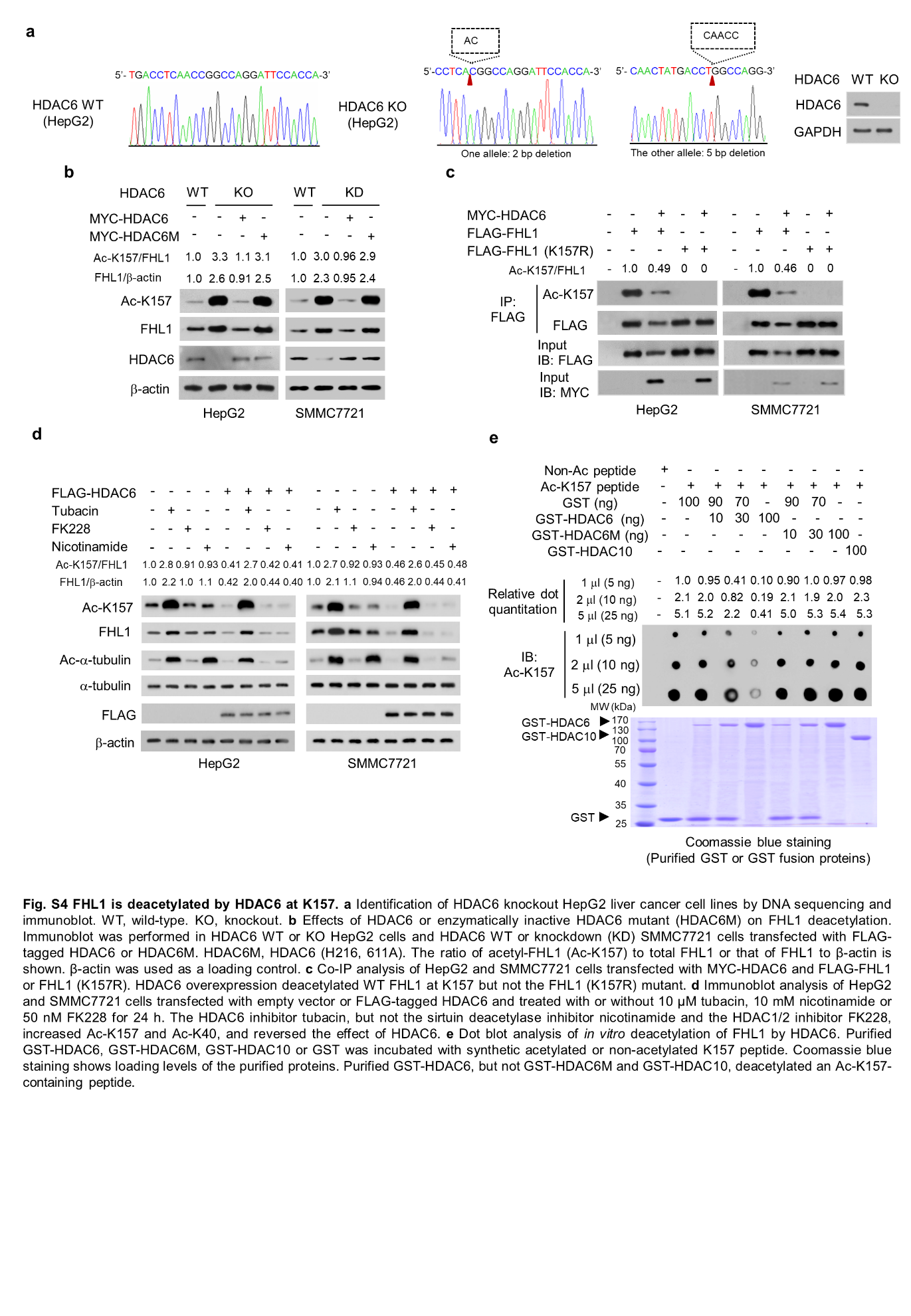


**Figure. S4 FHL1 is deacetylated by HDAC6 at K157. a** Identification of HDAC6 knockout HepG2 liver cancer cell lines by DNA sequencing and immunoblot. WT, wild-type. KO, knockout. **b** Effects of HDAC6 or enzymatically inactive HDAC6 mutant (HDAC6M) on FHL1 deacetylation. Immunoblot was performed in HDAC6 WT or KO HepG2 cells and HDAC6 WT or knockdown (KD) SMMC7721 cells transfected with FLAG-tagged HDAC6 or HDAC6M. HDAC6M, HDAC6 (H216, 611A). The ratio of acetyl-FHL1 (Ac-K157) to total FHL1 or that of FHL1 to β-actin is shown. β-actin was used as a loading control. **c** Co-IP analysis of HepG2 and SMMC7721 cells transfected with MYC-HDAC6 and FLAG-FHL1 or FHL1 (K157R). HDAC6 overexpression deacetylated WT FHL1 at K157 but not the FHL1 (K157R) mutant. **d** Immunoblot analysis of HepG2 and SMMC7721 cells transfected with empty vector or FLAG-tagged HDAC6 and treated with or without 10 μM tubacin, 10 mM nicotinamide or 50 nM FK228 for 24 h. The HDAC6 inhibitor tubacin, but not the sirtuin deacetylase inhibitor nicotinamide and the HDAC1/2 inhibitor FK228, increased Ac-K157 and Ac-K40, and reversed the effect of HDAC6. **e** Dot blot analysis of *in vitro* deacetylation of FHL1 by HDAC6. Purified GST-HDAC6, GST-HDAC6M, GST-HDAC10 or GST was incubated with synthetic acetylated or non-acetylated K157 peptide. Coomassie blue staining shows loading levels of the purified proteins. Purified GST-HDAC6, but not GST-HDAC6M and GST-HDAC10, deacetylated an Ac-K157-containing peptide.

Figure. S5.


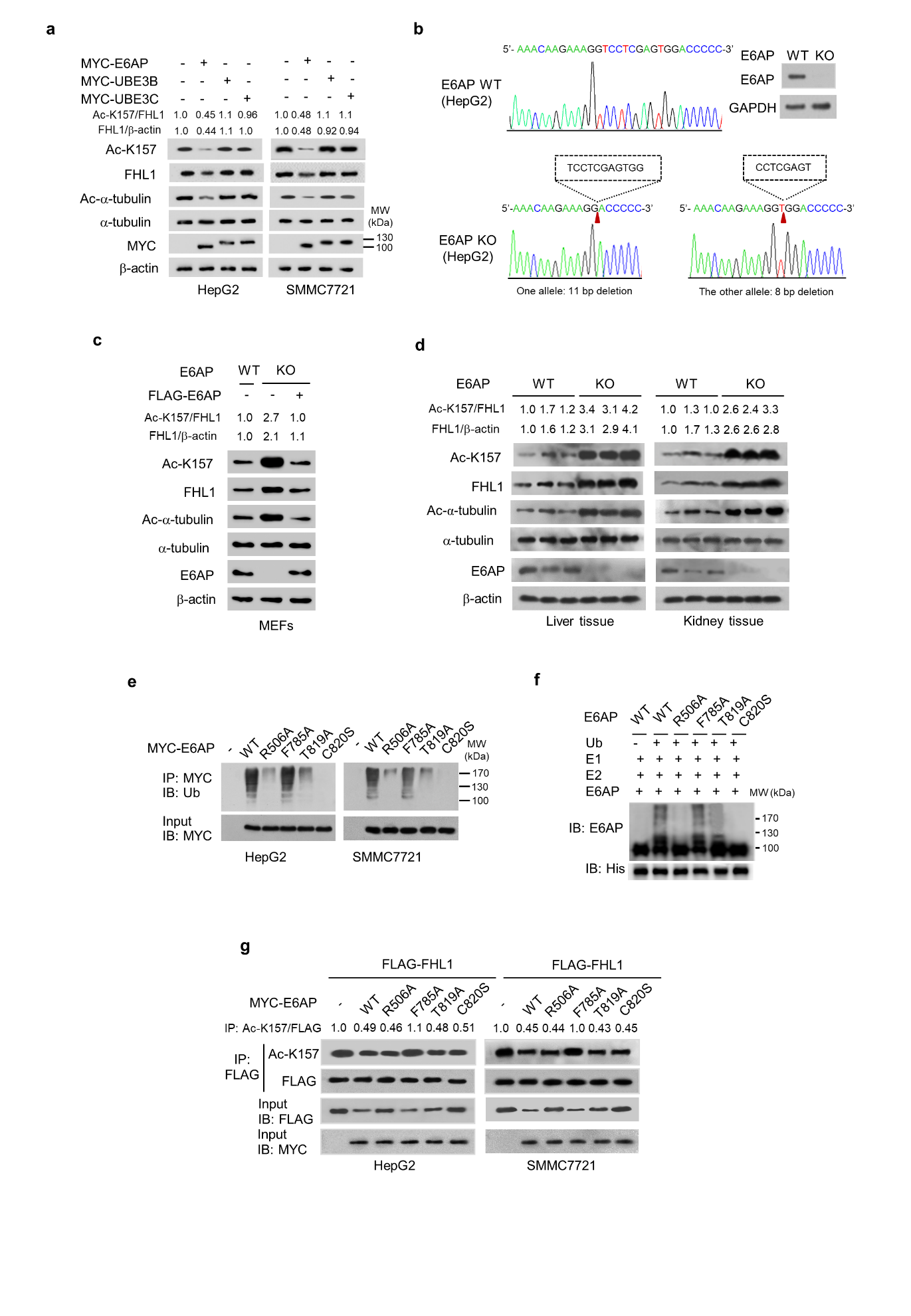


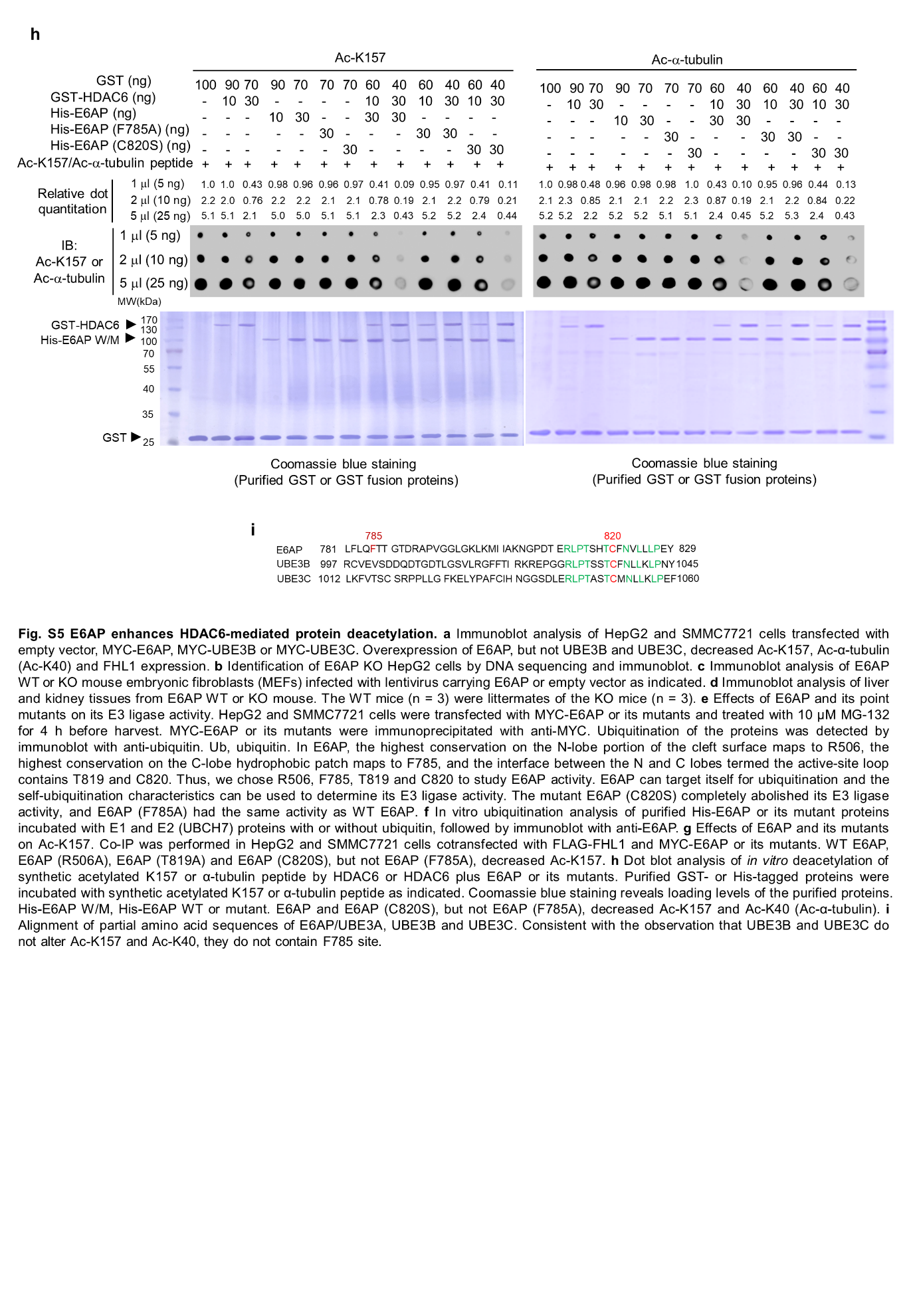


**Figure. S5 E6AP enhances HDAC6-mediated protein deacetylation. a** Immunoblot analysis of HepG2 and SMMC7721 cells transfected with empty vector, MYC-E6AP, MYC-UBE3B or MYC-UBE3C. Overexpression of E6AP, but not UBE3B and UBE3C, decreased Ac-K157, Ac-α-tubulin (Ac-K40) and FHL1 expression. **b** Identification of E6AP KO HepG2 cells by DNA sequencing and immunoblot. **c** Immunoblot analysis of E6AP WT or KO mouse embryonic fibroblasts (MEFs) infected with lentivirus carrying E6AP or empty vector as indicated. **d** Immunoblot analysis of liver and kidney tissues from E6AP WT or KO mouse. The WT mice (n = 3) were littermates of the KO mice (n = 3). **e** Effects of E6AP and its point mutants on its E3 ligase activity. HepG2 and SMMC7721 cells were transfected with MYC-E6AP or its mutants and treated with 10 μM MG-132 for 4 h before harvest. MYC-E6AP or its mutants were immunoprecipitated with anti-MYC. Ubiquitination of the proteins was detected by immunoblot with anti-ubiquitin. Ub, ubiquitin. In E6AP, the highest conservation on the N-lobe portion of the cleft surface maps to R506, the highest conservation on the C-lobe hydrophobic patch maps to F785, and the interface between the N and C lobes termed the active-site loop contains T819 and C820. Thus, we chose R506, F785, T819 and C820 to study E6AP activity. E6AP can target itself for ubiquitination and the self-ubiquitination characteristics can be used to determine its E3 ligase activity. The mutant E6AP (C820S) completely abolished its E3 ligase activity, and E6AP (F785A) had the same activity as WT E6AP. **f** In vitro ubiquitination analysis of purified His-E6AP or its mutant proteins incubated with E1 and E2 (UBCH7) proteins with or without ubiquitin, followed by immunoblot with anti-E6AP. **g** Effects of E6AP and its mutants on Ac-K157. Co-IP was performed in HepG2 and SMMC7721 cells cotransfected with FLAG-FHL1 and MYC-E6AP or its mutants. WT E6AP, E6AP (R506A), E6AP (T819A) and E6AP (C820S), but not E6AP (F785A), decreased Ac-K157. **h** Dot blot analysis of *in vitro* deacetylation of synthetic acetylated K157 or α-tubulin peptide by HDAC6 or HDAC6 plus E6AP or its mutants. Purified GST- or His-tagged proteins were incubated with synthetic acetylated K157 or α-tubulin peptide as indicated. Coomassie blue staining reveals loading levels of the purified proteins. His-E6AP W/M, His-E6AP WT or mutant. E6AP and E6AP (C820S), but not E6AP (F785A), decreased Ac-K157 and Ac-K40 (Ac-α-tubulin). **i** Alignment of partial amino acid sequences of E6AP/UBE3A, UBE3B and UBE3C. Consistent with the observation that UBE3B and UBE3C do not alter Ac-K157 and Ac-K40, they do not contain F785 site.

Figure. S6.


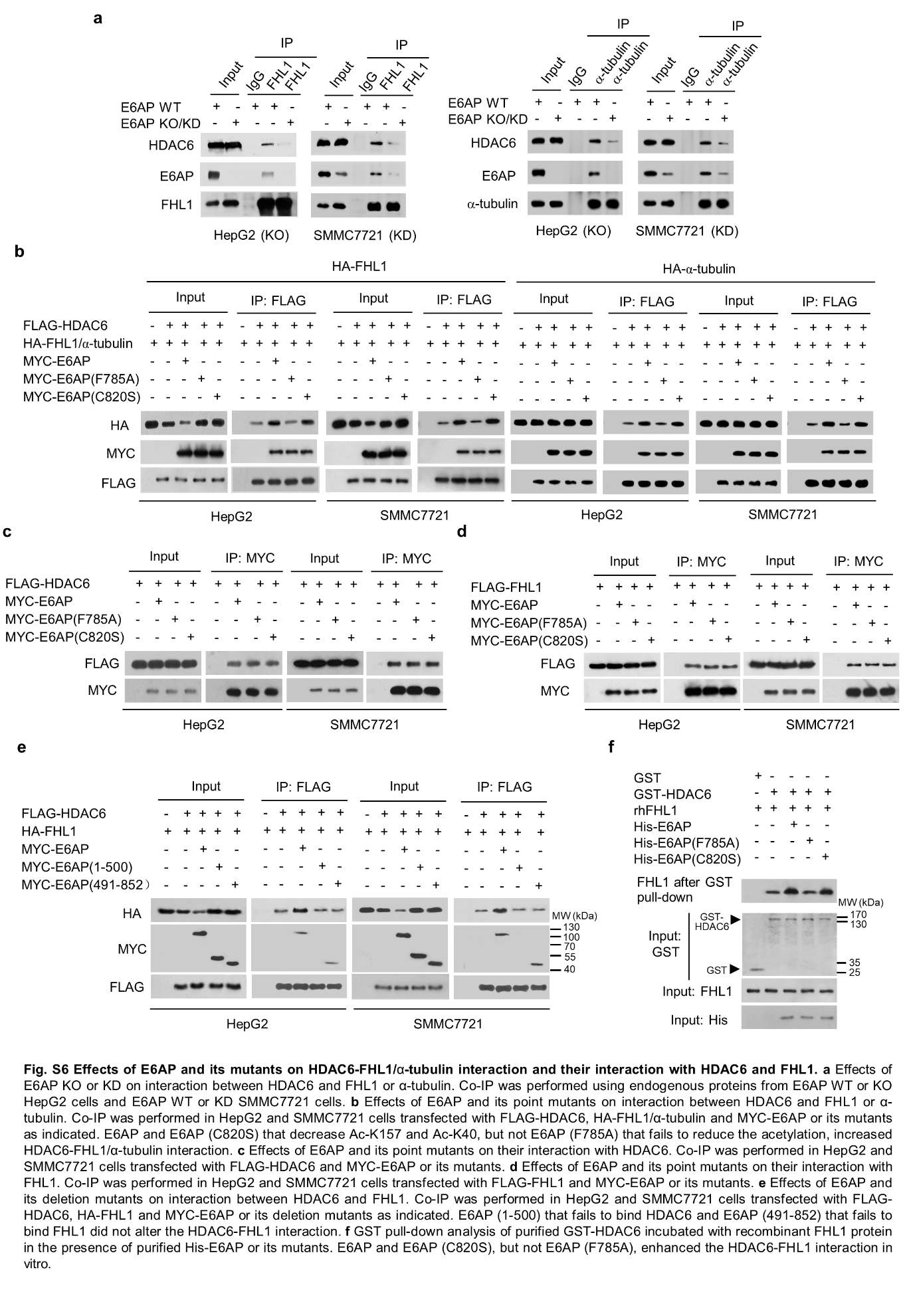


**Figure. S6 Effects of E6AP and its mutants on HDAC6-FHL1/**α**-tubulin interaction and their interaction with HDAC6 and FHL1. a** Effects of E6AP KO or KD on interaction between HDAC6 and FHL1 or α-tubulin. Co-IP was performed using endogenous proteins from E6AP WT or KO HepG2 cells and E6AP WT or KD SMMC7721 cells. **b** Effects of E6AP and its point mutants on interaction between HDAC6 and FHL1 or α-tubulin. Co-IP was performed in HepG2 and SMMC7721 cells transfected with FLAG-HDAC6, HA-FHL1/α-tubulin and MYC-E6AP or its mutants as indicated. E6AP and E6AP (C820S) that decrease Ac-K157 and Ac-K40, but not E6AP (F785A) that fails to reduce the acetylation, increased HDAC6-FHL1/α-tubulin interaction. **c** Effects of E6AP and its point mutants on their interaction with HDAC6. Co-IP was performed in HepG2 and SMMC7721 cells transfected with FLAG-HDAC6 and MYC-E6AP or its mutants. **d** Effects of E6AP and its point mutants on their interaction with FHL1. Co-IP was performed in HepG2 and SMMC7721 cells transfected with FLAG-FHL1 and MYC-E6AP or its mutants. **e** Effects of E6AP and its deletion mutants on interaction between HDAC6 and FHL1. Co-IP was performed in HepG2 and SMMC7721 cells transfected with FLAG-HDAC6, HA-FHL1 and MYC-E6AP or its deletion mutants as indicated. E6AP (1-500) that fails to bind HDAC6 and E6AP (491-852) that fails to bind FHL1 did not alter the HDAC6-FHL1 interaction. **f** GST pull-down analysis of purified GST-HDAC6 incubated with recombinant FHL1 protein in the presence of purified His-E6AP or its mutants. E6AP and E6AP (C820S), but not E6AP (F785A), enhanced the HDAC6-FHL1 interaction in vitro.

Figure. S7.


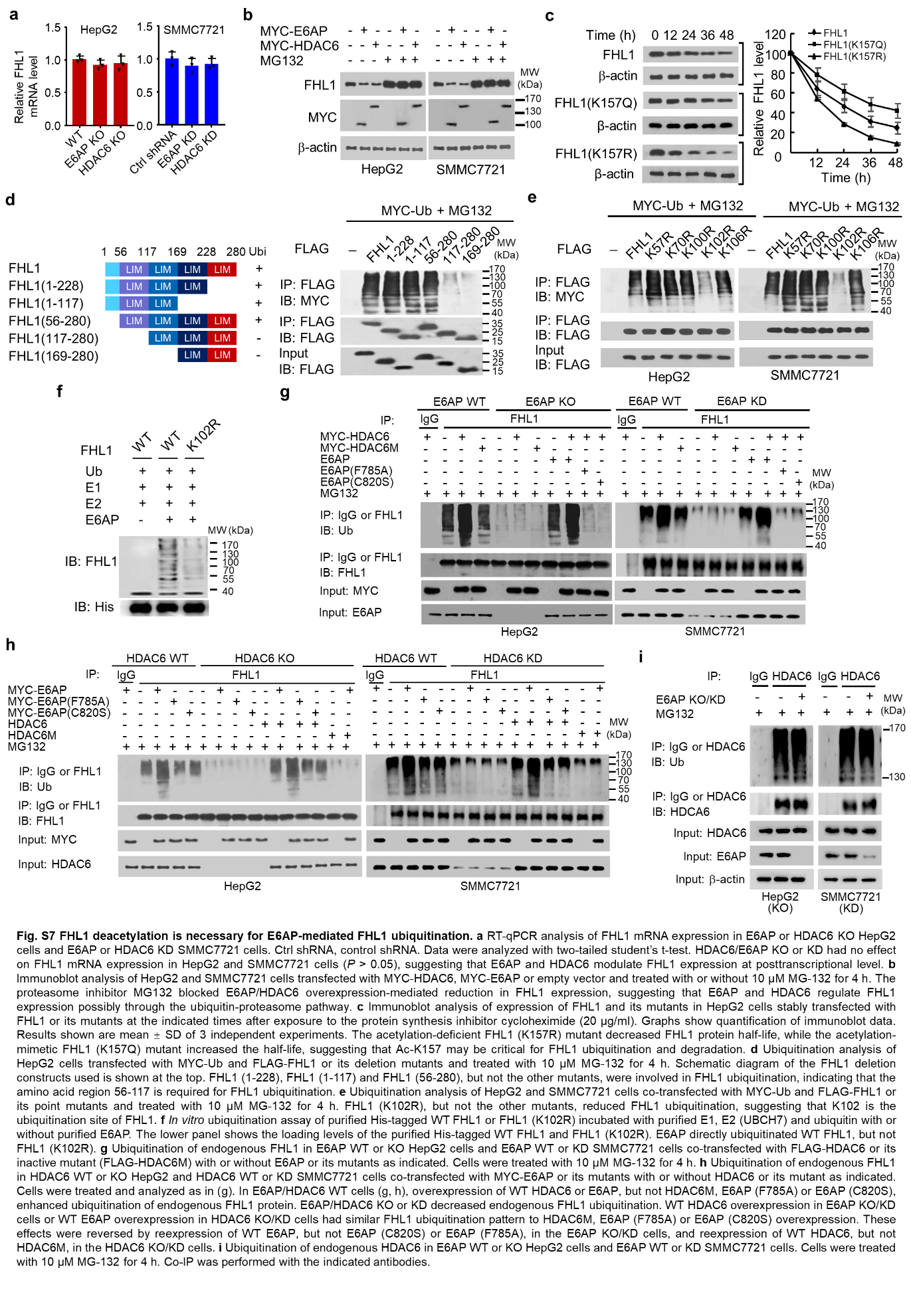


**Figure. S7 FHL1 deacetylation is necessary for E6AP-mediated FHL1 ubiquitination. a** RT-qPCR analysis of FHL1 mRNA expression in E6AP or HDAC6 KO HepG2 cells and E6AP or HDAC6 KD SMMC7721 cells. Ctrl shRNA, control shRNA. Data were analyzed with two-tailed student’s t-test. HDAC6/E6AP KO or KD had no effect on FHL1 mRNA expression in HepG2 and SMMC7721 cells (*P* > 0.05), suggesting that E6AP and HDAC6 modulate FHL1 expression at posttranscriptional level. **b** Immunoblot analysis of HepG2 and SMMC7721 cells transfected with MYC-HDAC6, MYC-E6AP or empty vector and treated with or without 10 μM MG-132 for 4 h. The proteasome inhibitor MG132 blocked E6AP/HDAC6 overexpression-mediated reduction in FHL1 expression, suggesting that E6AP and HDAC6 regulate FHL1 expression possibly through the ubiquitin-proteasome pathway. **c** Immunoblot analysis of expression of FHL1 and its mutants in HepG2 cells stably transfected with FHL1 or its mutants at the indicated times after exposure to the protein synthesis inhibitor cycloheximide (20 μg/ml). Graphs show quantification of immunoblot data. Results shown are mean ± SD of 3 independent experiments. The acetylation-deficient FHL1 (K157R) mutant decreased FHL1 protein half-life, while the acetylation-mimetic FHL1 (K157Q) mutant increased the half-life, suggesting that Ac-K157 may be critical for FHL1 ubiquitination and degradation. **d** Ubiquitination analysis of HepG2 cells transfected with MYC-Ub and FLAG-FHL1 or its deletion mutants and treated with 10 μM MG-132 for 4 h. Schematic diagram of the FHL1 deletion constructs used is shown at the top. FHL1 (1-228), FHL1 (1-117) and FHL1 (56-280), but not the other mutants, were involved in FHL1 ubiquitination, indicating that the amino acid region 56-117 is required for FHL1 ubiquitination. **e** Ubiquitination analysis of HepG2 and SMMC7721 cells co-transfected with MYC-Ub and FLAG-FHL1 or its point mutants and treated with 10 μM MG-132 for 4 h. FHL1 (K102R), but not the other mutants, reduced FHL1 ubiquitination, suggesting that K102 is the ubiquitination site of FHL1. **f** *In vitro* ubiquitination assay of purified His-tagged WT FHL1 or FHL1 (K102R) incubated with purified E1, E2 (UBCH7) and ubiquitin with or without purified E6AP. The lower panel shows the loading levels of the purified His-tagged WT FHL1 and FHL1 (K102R). E6AP directly ubiquitinated WT FHL1, but not FHL1 (K102R). **g** Ubiquitination of endogenous FHL1 in E6AP WT or KO HepG2 cells and E6AP WT or KD SMMC7721 cells co-transfected with FLAG-HDAC6 or its inactive mutant (FLAG-HDAC6M) with or without E6AP or its mutants as indicated. Cells were treated with 10 μM MG-132 for 4 h. **h** Ubiquitination of endogenous FHL1 in HDAC6 WT or KO HepG2 and HDAC6 WT or KD SMMC7721 cells co-transfected with MYC-E6AP or its mutants with or without HDAC6 or its mutant as indicated. Cells were treated and analyzed as in (g). In E6AP/HDAC6 WT cells (g, h), overexpression of WT HDAC6 or E6AP, but not HDAC6M, E6AP (F785A) or E6AP (C820S), enhanced ubiquitination of endogenous FHL1 protein. E6AP/HDAC6 KO or KD decreased endogenous FHL1 ubiquitination. WT HDAC6 overexpression in E6AP KO/KD cells or WT E6AP overexpression in HDAC6 KO/KD cells had similar FHL1 ubiquitination pattern to HDAC6M, E6AP (F785A) or E6AP (C820S) overexpression. These effects were reversed by reexpression of WT E6AP, but not E6AP (C820S) or E6AP (F785A), in the E6AP KO/KD cells, and reexpression of WT HDAC6, but not HDAC6M, in the HDAC6 KO/KD cells. **i** Ubiquitination of endogenous HDAC6 in E6AP WT or KO HepG2 cells and E6AP WT or KD SMMC7721 cells. Cells were treated with 10 μM MG-132 for 4 h. Co-IP was performed with the indicated antibodies.

Figure. S8.


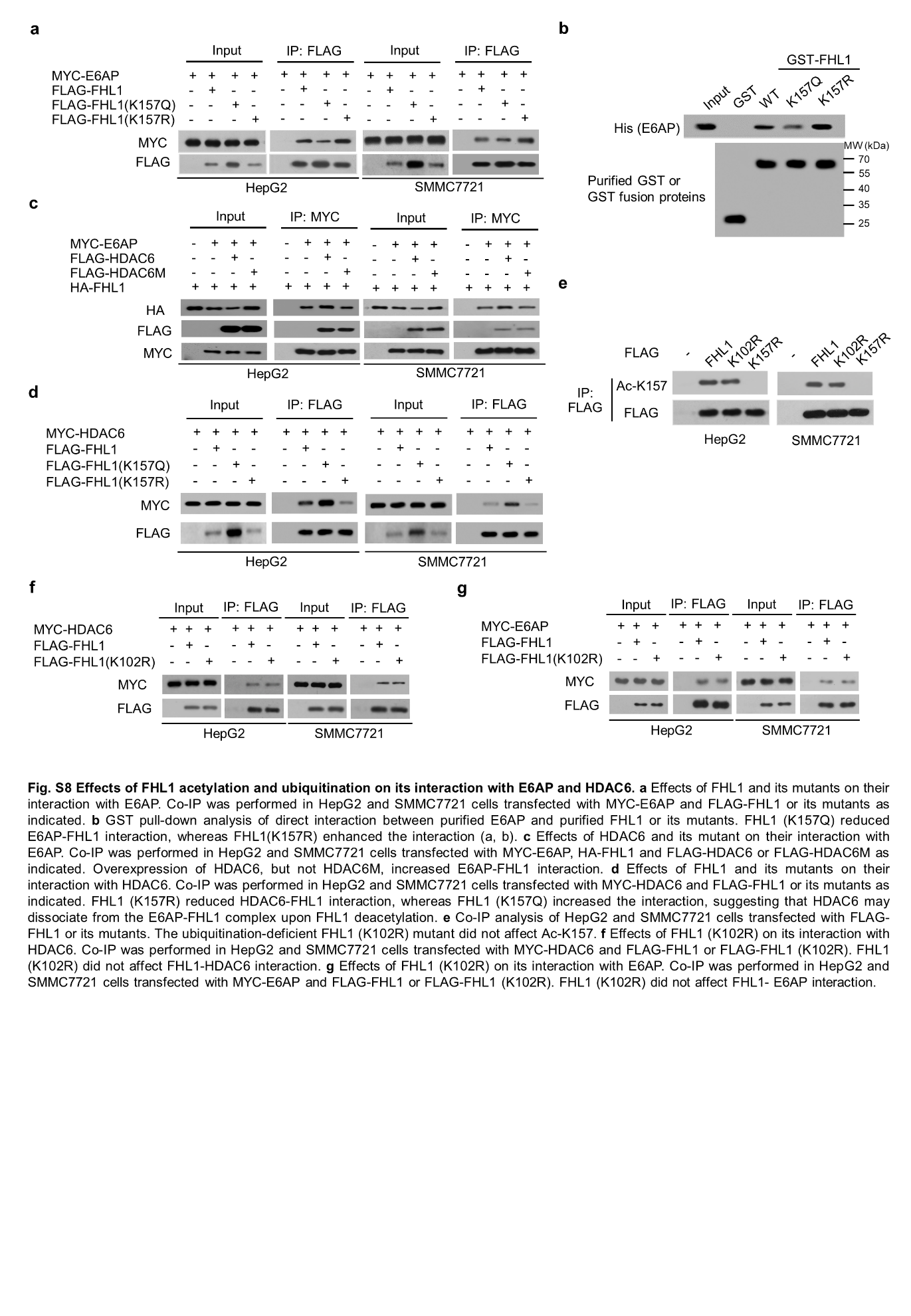


**Figure. S8 Effects of FHL1 acetylation and ubiquitination on its interaction with E6AP and HDAC6. a** Effects of FHL1 and its mutants on their interaction with E6AP. Co-IP was performed in HepG2 and SMMC7721 cells transfected with MYC-E6AP and FLAG-FHL1 or its mutants as indicated. **b** GST pull-down analysis of direct interaction between purified E6AP and purified FHL1 or its mutants. FHL1 (K157Q) reduced E6AP-FHL1 interaction, whereas FHL1(K157R) enhanced the interaction (a, b). **c** Effects of HDAC6 and its mutant on their interaction with E6AP. Co-IP was performed in HepG2 and SMMC7721 cells transfected with MYC-E6AP, HA-FHL1 and FLAG-HDAC6 or FLAG-HDAC6M as indicated. Overexpression of HDAC6, but not HDAC6M, increased E6AP-FHL1 interaction. **d** Effects of FHL1 and its mutants on their interaction with HDAC6. Co-IP was performed in HepG2 and SMMC7721 cells transfected with MYC-HDAC6 and FLAG-FHL1 or its mutants as indicated. FHL1 (K157R) reduced HDAC6-FHL1 interaction, whereas FHL1 (K157Q) increased the interaction, suggesting that HDAC6 may dissociate from the E6AP-FHL1 complex upon FHL1 deacetylation. **e** Co-IP analysis of HepG2 and SMMC7721 cells transfected with FLAG-FHL1 or its mutants. The ubiquitination-deficient FHL1 (K102R) mutant did not affect Ac-K157. **f** Effects of FHL1 (K102R) on its interaction with HDAC6. Co-IP was performed in HepG2 and SMMC7721 cells transfected with MYC-HDAC6 and FLAG-FHL1 or FLAG-FHL1 (K102R). FHL1 (K102R) did not affect FHL1-HDAC6 interaction. **g** Effects of FHL1 (K102R) on its interaction with E6AP. Co-IP was performed in HepG2 and SMMC7721 cells transfected with MYC-E6AP and FLAG-FHL1 or FLAG-FHL1 (K102R). FHL1 (K102R) did not affect FHL1- E6AP interaction.

Figure. S9.


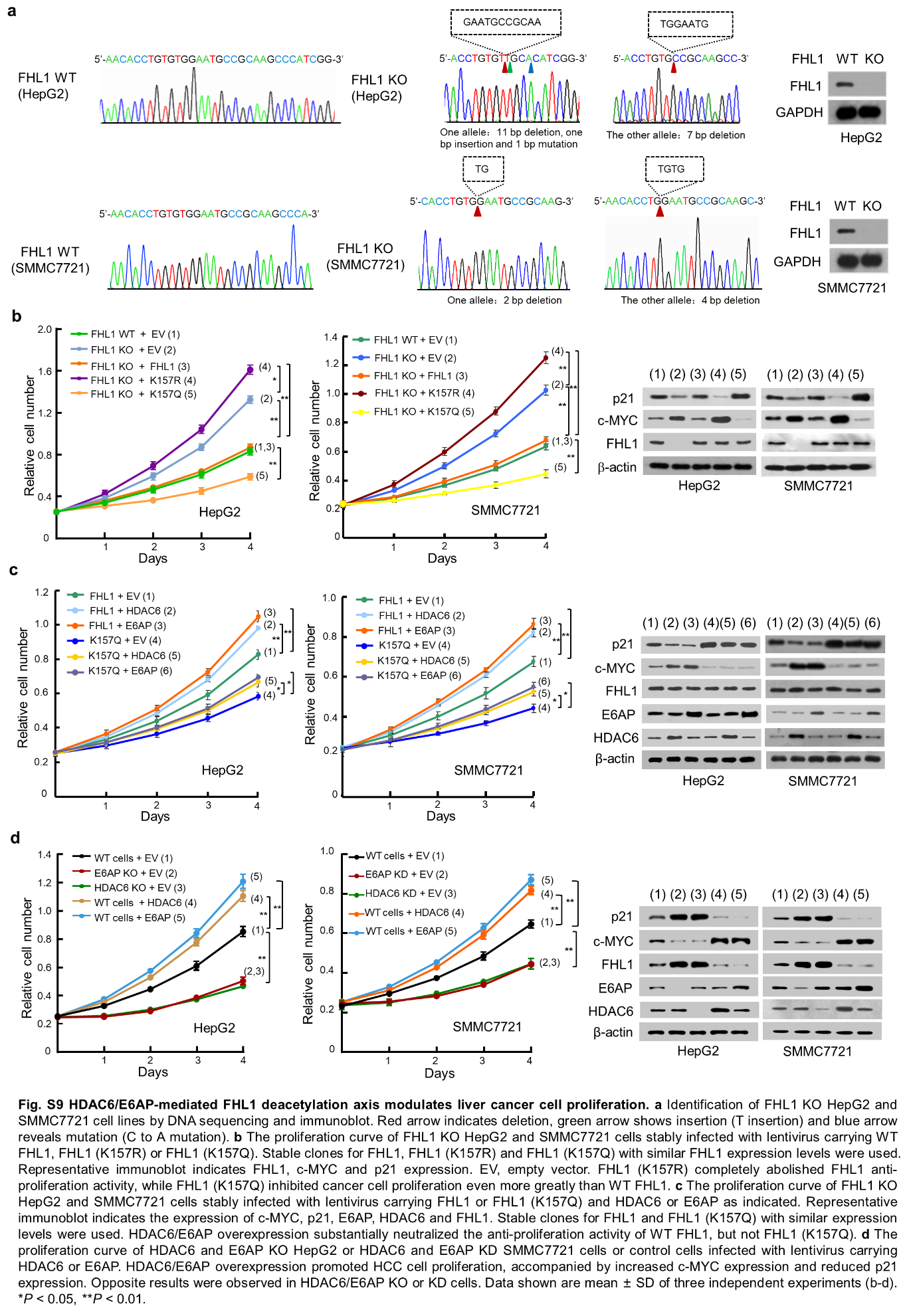


**Figure. S9 HDAC6/E6AP-mediated FHL1 deacetylation axis modulates liver cancer cell proliferation. a** Identification of FHL1 KO HepG2 and SMMC7721 cell lines by DNA sequencing and immunoblot. Red arrow indicates deletion, green arrow shows insertion (T insertion) and blue arrow reveals mutation (C to A mutation). **b** The proliferation curve of FHL1 KO HepG2 and SMMC7721 cells stably infected with lentivirus carrying WT FHL1, FHL1 (K157R) or FHL1 (K157Q). Stable clones for FHL1, FHL1 (K157R) and FHL1 (K157Q) with similar FHL1 expression levels were used. Representative immunoblot indicates FHL1, c-MYC and p21 expression. EV, empty vector. FHL1 (K157R) completely abolished FHL1 anti-proliferation activity, while FHL1 (K157Q) inhibited cancer cell proliferation even more greatly than WT FHL1. **c** The proliferation curve of FHL1 KO HepG2 and SMMC7721 cells stably infected with lentivirus carrying FHL1 or FHL1 (K157Q) and HDAC6 or E6AP as indicated. Representative immunoblot indicates the expression of c-MYC, p21, E6AP, HDAC6 and FHL1. Stable clones for FHL1 and FHL1 (K157Q) with similar expression levels were used. HDAC6/E6AP overexpression substantially neutralized the anti-proliferation activity of WT FHL1, but not FHL1 (K157Q). **d** The proliferation curve of HDAC6 and E6AP KO HepG2 or HDAC6 and E6AP KD SMMC7721 cells or control cells infected with lentivirus carrying HDAC6 or E6AP. HDAC6/E6AP overexpression promoted HCC cell proliferation, accompanied by increased c-MYC expression and reduced p21 expression. Opposite results were observed in HDAC6/E6AP KO or KD cells. Data shown are mean ± SD of three independent experiments (b-d). **P* < 0.05, ***P* < 0.01.

Figure. S10.


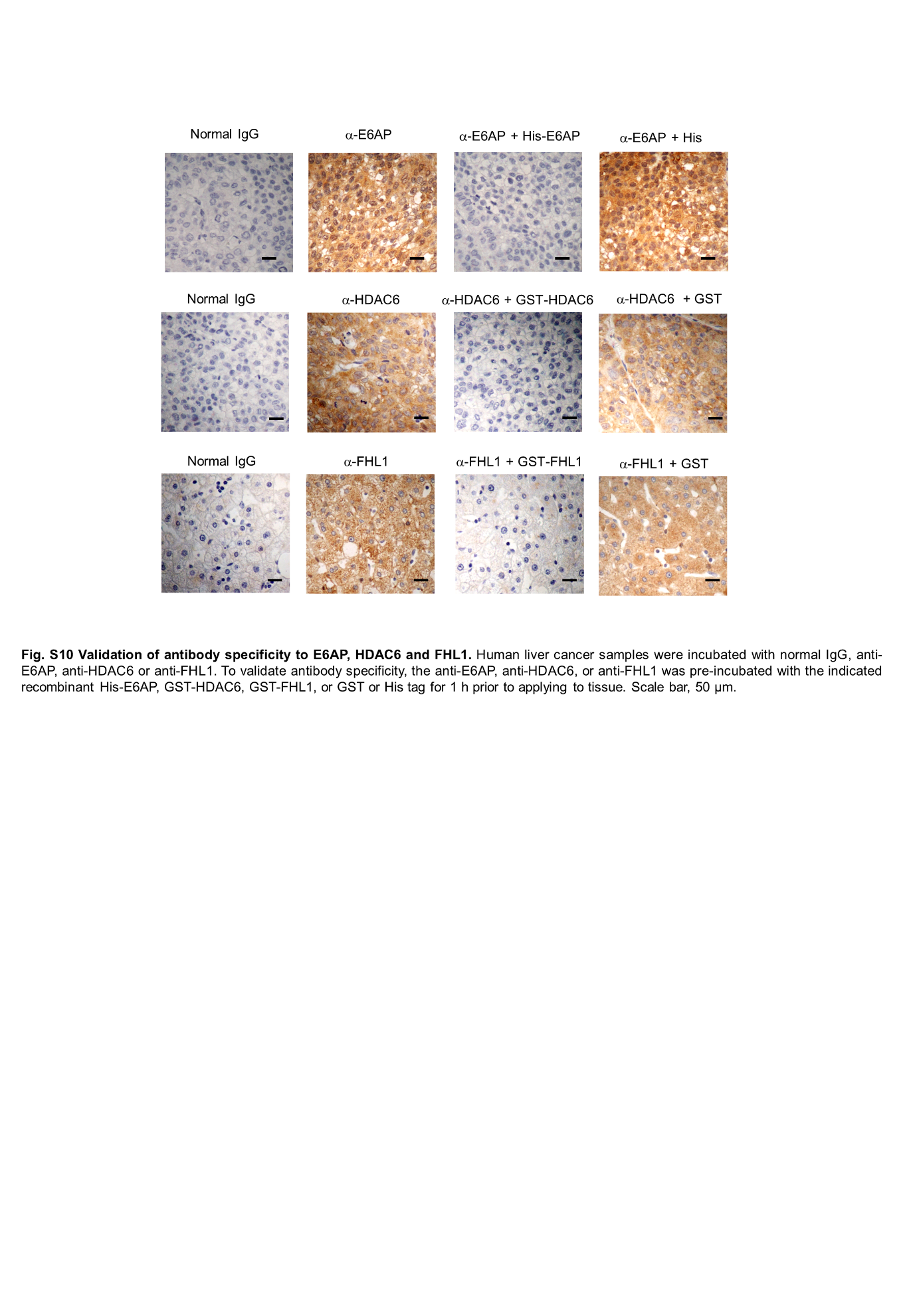


**Figure. S10 Validation of antibody specificity to E6AP, HDAC6 and FHL1.** Human liver cancer samples were incubated with normal IgG, anti-E6AP, anti-HDAC6 or anti-FHL1. To validate antibody specificity, the anti-E6AP, anti-HDAC6, or anti-FHL1 was pre-incubated with the indicated recombinant His-E6AP, GST-HDAC6, GST-FHL1, or GST or His tag for 1 h prior to applying to tissue. Scale bar, 50 μm.

Figure. S11.


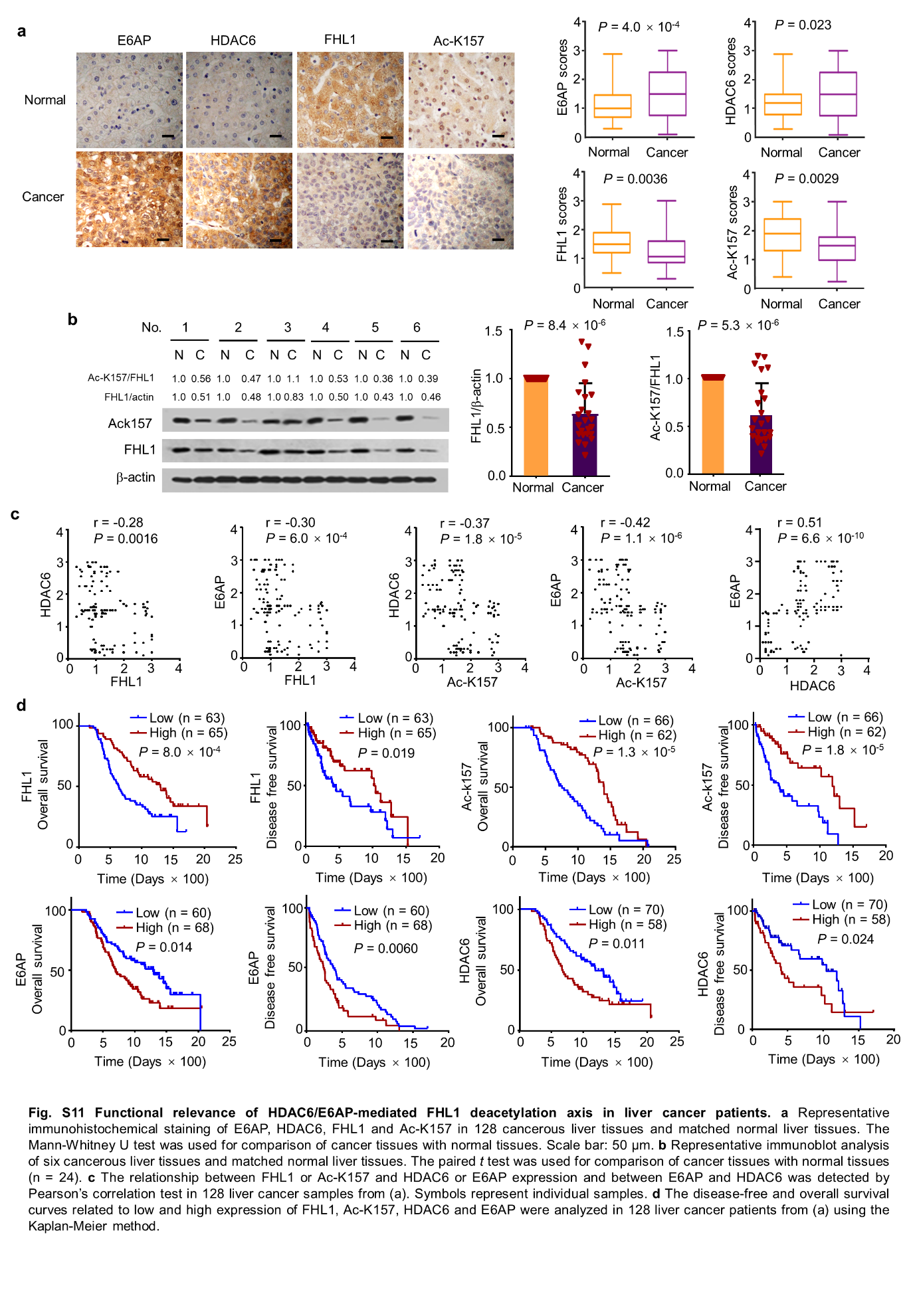


**Figure. S11 Functional relevance of HDAC6/E6AP-mediated FHL1 deacetylation axis in liver cancer patients. a** Representative immunohistochemical staining of E6AP, HDAC6, FHL1 and Ac-K157 in 128 cancerous liver tissues and matched normal liver tissues. The Mann-Whitney U test was used for comparison of cancer tissues with normal tissues. Scale bar: 50 μm. **b** Representative immunoblot analysis of six cancerous liver tissues and matched normal liver tissues. The paired *t* test was used for comparison of cancer tissues with normal tissues (n = 24). **c** The relationship between FHL1 or Ac-K157 and HDAC6 or E6AP expression and between E6AP and HDAC6 was detected by Pearson’s correlation test in 128 liver cancer samples from (a). Symbols represent individual samples. **d** The disease-free and overall survival curves related to low and high expression of FHL1, Ac-K157, HDAC6 and E6AP were analyzed in 128 liver cancer patients from (a) using the Kaplan-Meier method.

Figure. S12.


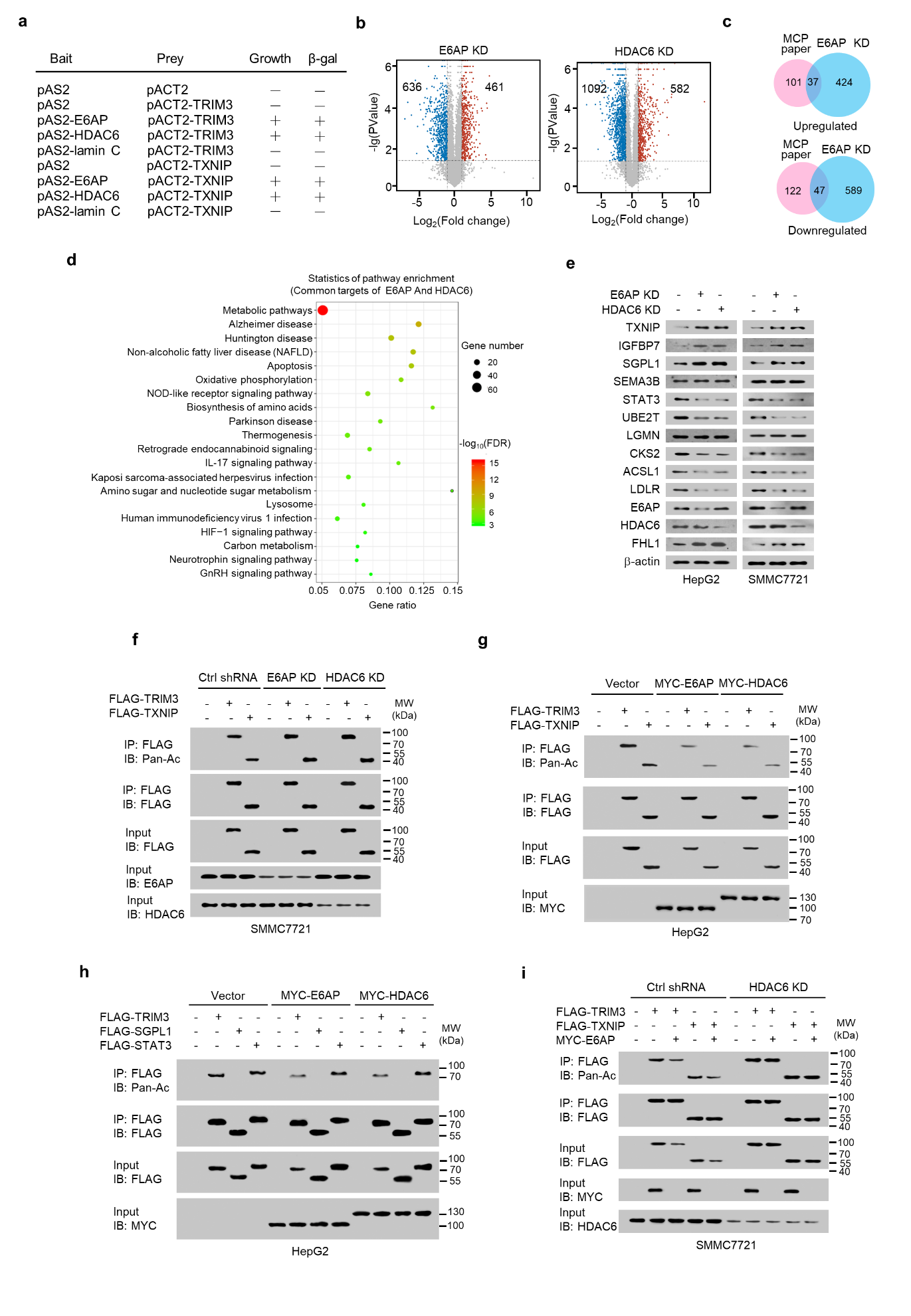


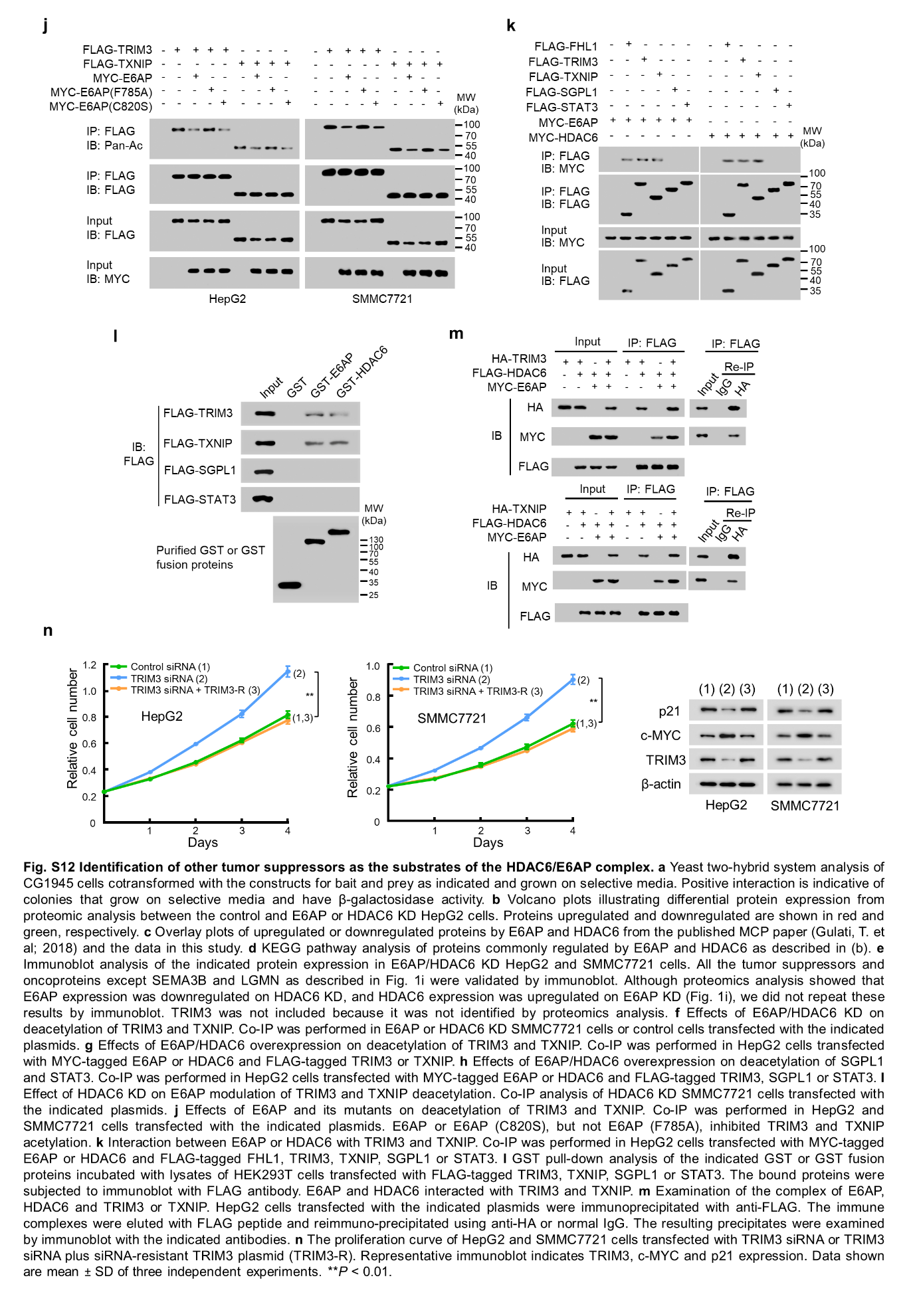


**Figure. S12 Identification of other tumor suppressors as the substrates of the HDAC6/E6AP complex. a** Yeast two-hybrid system analysis of CG1945 cells cotransformed with the constructs for bait and prey as indicated and grown on selective media. Positive interaction is indicative of colonies that grow on selective media and have β-galactosidase activity. **b** Volcano plots illustrating differential protein expression from proteomic analysis between the control and E6AP or HDAC6 KD HepG2 cells. Proteins upregulated and downregulated are shown in red and green, respectively. **c** Overlay plots of upregulated or downregulated proteins by E6AP and HDAC6 from the published MCP paper (Gulati, T. et al; 2018) and the data in this study. **d** KEGG pathway analysis of proteins commonly regulated by E6AP and HDAC6 as described in (b). **e** Immunoblot analysis of the indicated protein expression in E6AP/HDAC6 KD HepG2 and SMMC7721 cells. All the tumor suppressors and oncoproteins except SEMA3B and LGMN as described in Fig. 1i were validated by immunoblot. Although proteomics analysis showed that E6AP expression was downregulated on HDAC6 KD, and HDAC6 expression was upregulated on E6AP KD (Fig. 1i), we did not repeat these results by immunoblot. TRIM3 was not included because it was not identified by proteomics analysis. **f** Effects of E6AP/HDAC6 KD on deacetylation of TRIM3 and TXNIP. Co-IP was performed in E6AP or HDAC6 KD SMMC7721 cells or control cells transfected with the indicated plasmids. **g** Effects of E6AP/HDAC6 overexpression on deacetylation of TRIM3 and TXNIP. Co-IP was performed in HepG2 cells transfected with MYC-tagged E6AP or HDAC6 and FLAG-tagged TRIM3 or TXNIP. **h** Effects of E6AP/HDAC6 overexpression on deacetylation of SGPL1 and STAT3. Co-IP was performed in HepG2 cells transfected with MYC-tagged E6AP or HDAC6 and FLAG-tagged TRIM3, SGPL1 or STAT3. **i** Effect of HDAC6 KD on E6AP modulation of TRIM3 and TXNIP deacetylation. Co-IP analysis of HDAC6 KD SMMC7721 cells transfected with the indicated plasmids. **j** Effects of E6AP and its mutants on deacetylation of TRIM3 and TXNIP. Co-IP was performed in HepG2 and SMMC7721 cells transfected with the indicated plasmids. E6AP or E6AP (C820S), but not E6AP (F785A), inhibited TRIM3 and TXNIP acetylation. **k** Interaction between E6AP or HDAC6 with TRIM3 and TXNIP. Co-IP was performed in HepG2 cells transfected with MYC-tagged E6AP or HDAC6 and FLAG-tagged FHL1, TRIM3, TXNIP, SGPL1 or STAT3. **l** GST pull-down analysis of the indicated GST or GST fusion proteins incubated with lysates of HEK293T cells transfected with FLAG-tagged TRIM3, TXNIP, SGPL1 or STAT3. The bound proteins were subjected to immunoblot with FLAG antibody. E6AP and HDAC6 interacted with TRIM3 and TXNIP. **m** Examination of the complex of E6AP, HDAC6 and TRIM3 or TXNIP. HepG2 cells transfected with the indicated plasmids were immunoprecipitated with anti-FLAG. The immune complexes were eluted with FLAG peptide and reimmuno-precipitated using anti-HA or normal IgG. The resulting precipitates were examined by immunoblot with the indicated antibodies. **n** The proliferation curve of HepG2 and SMMC7721 cells transfected with TRIM3 siRNA or TRIM3 siRNA plus siRNA-resistant TRIM3 plasmid (TRIM3-R). Representative immunoblot indicates TRIM3, c-MYC and p21 expression. Data shown are mean ± SD of three independent experiments. ***P* < 0.01.

Figure. S13.


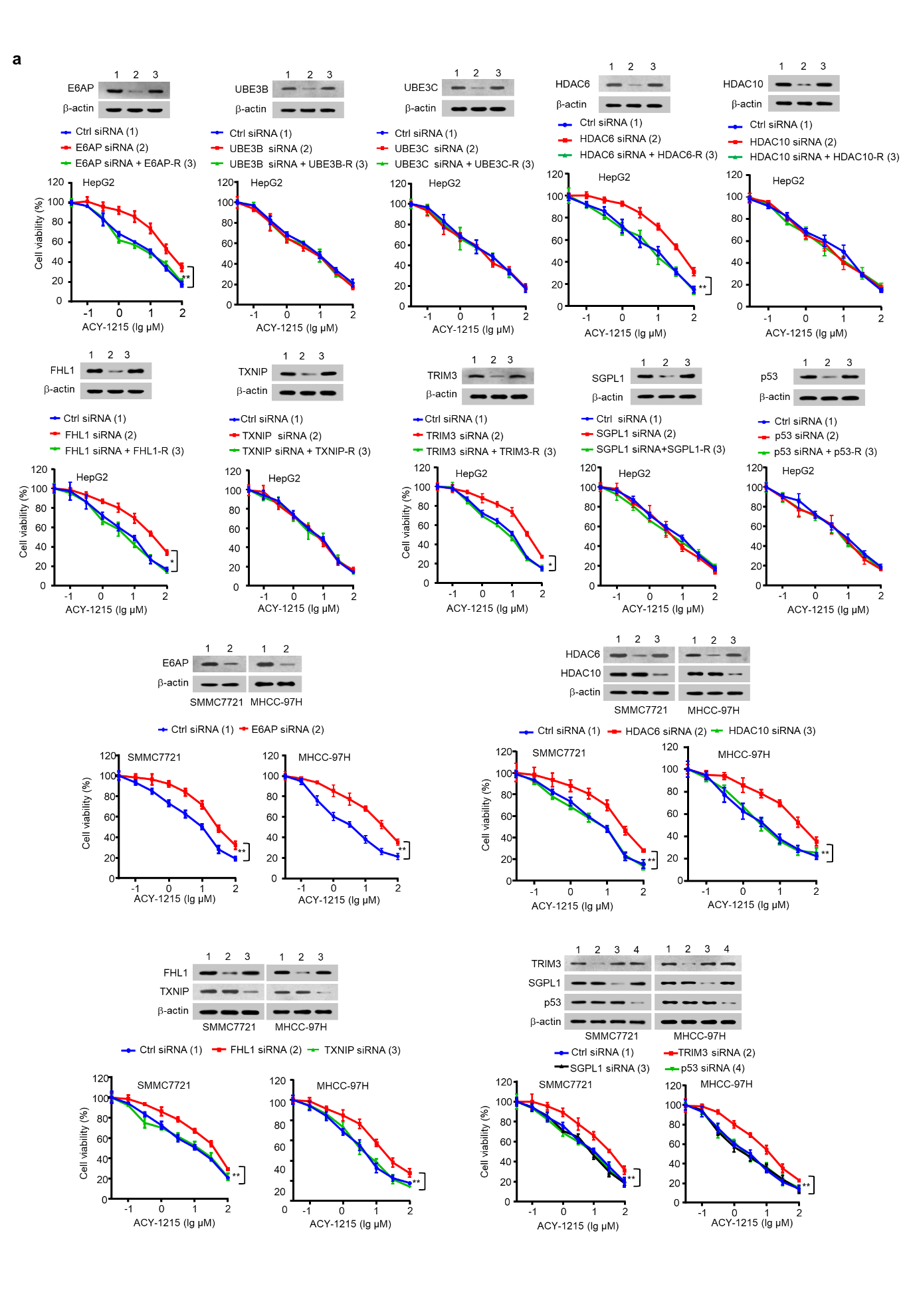


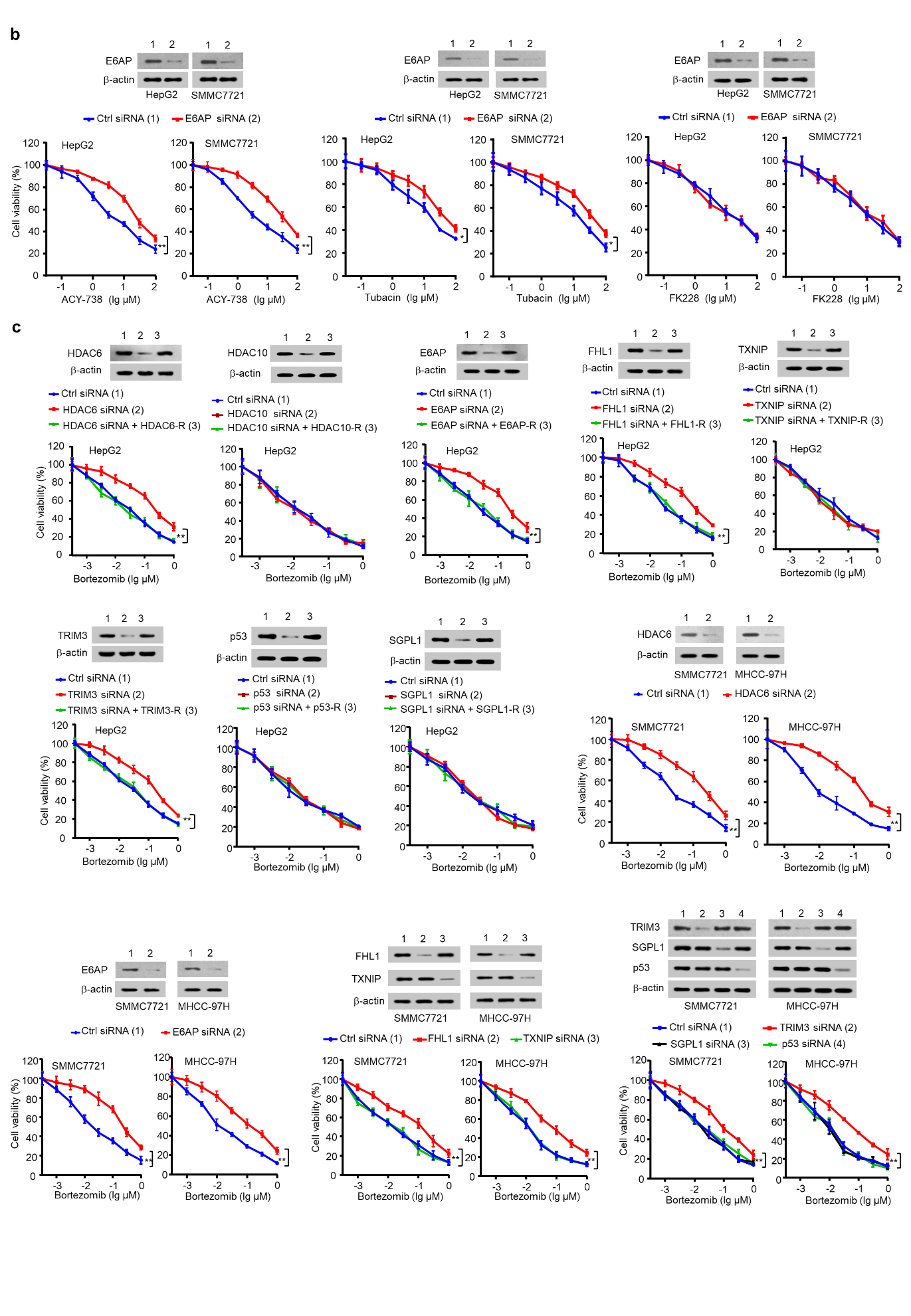

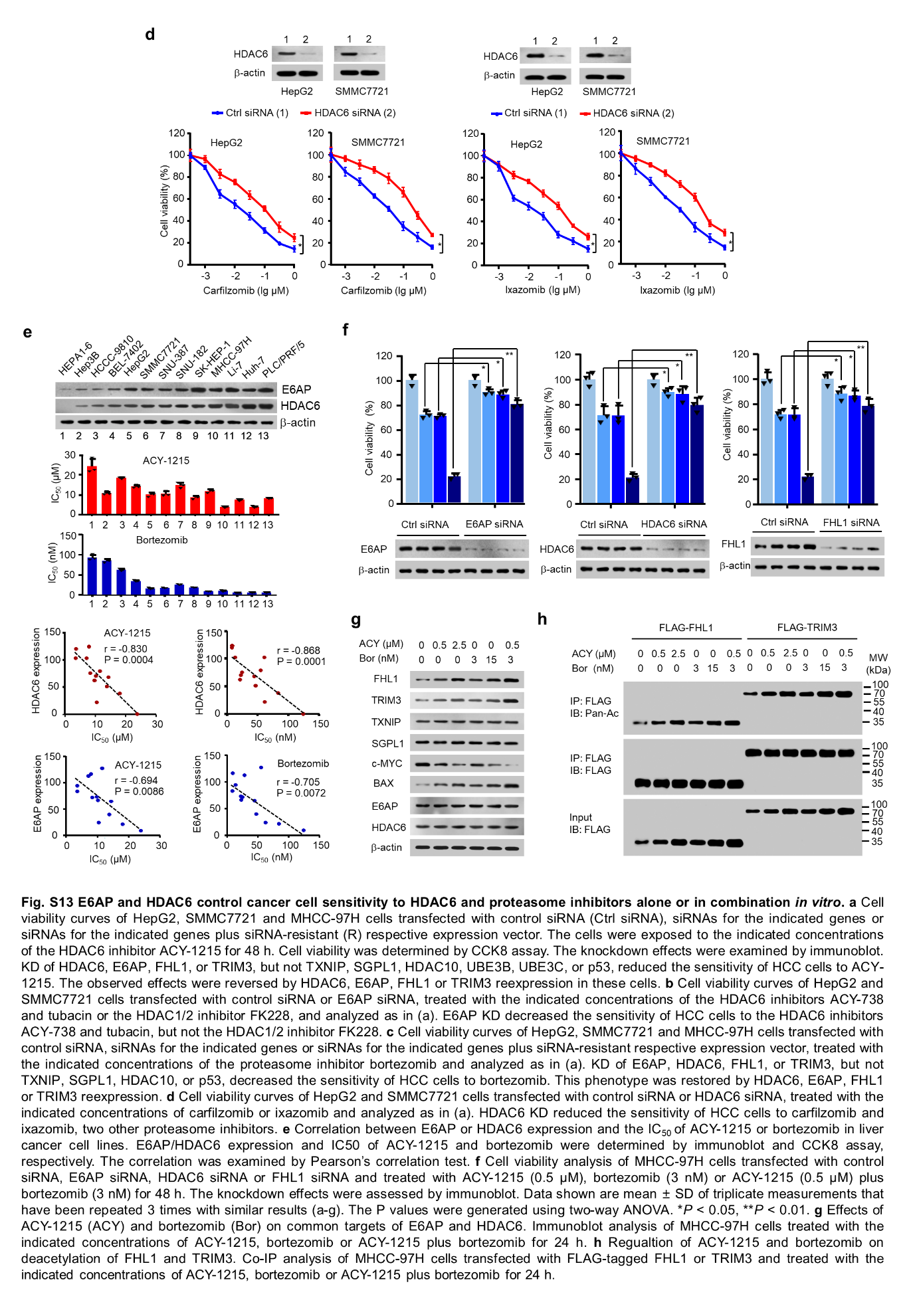


**Figure. S13 E6AP and HDAC6 control cancer cell sensitivity to HDAC6 and proteasome inhibitors alone or in combination *in vitro*. a** Cell viability curves of HepG2, SMMC7721 and MHCC-97H cells transfected with control siRNA (Ctrl siRNA), siRNAs for the indicated genes or siRNAs for the indicated genes plus siRNA-resistant (R) respective expression vector. The cells were exposed to the indicated concentrations of the HDAC6 inhibitor ACY-1215 for 48 h. Cell viability was determined by CCK8 assay. The knockdown effects were examined by immunoblot. KD of HDAC6, E6AP, FHL1, or TRIM3, but not TXNIP, SGPL1, HDAC10, UBE3B, UBE3C, or p53, reduced the sensitivity of HCC cells to ACY-1215. The observed effects were reversed by HDAC6, E6AP, FHL1 or TRIM3 reexpression in these cells. **b** Cell viability curves of HepG2 and SMMC7721 cells transfected with control siRNA or E6AP siRNA, treated with the indicated concentrations of the HDAC6 inhibitors ACY-738 and tubacin or the HDAC1/2 inhibitor FK228, and analyzed as in (a). E6AP KD decreased the sensitivity of HCC cells to the HDAC6 inhibitors ACY-738 and tubacin, but not the HDAC1/2 inhibitor FK228. **c** Cell viability curves of HepG2, SMMC7721 and MHCC-97H cells transfected with control siRNA, siRNAs for the indicated genes or siRNAs for the indicated genes plus siRNA-resistant respective expression vector, treated with the indicated concentrations of the proteasome inhibitor bortezomib and analyzed as in (a). KD of E6AP, HDAC6, FHL1, or TRIM3, but not TXNIP, SGPL1, HDAC10, or p53, decreased the sensitivity of HCC cells to bortezomib. This phenotype was restored by HDAC6, E6AP, FHL1 or TRIM3 reexpression. **d** Cell viability curves of HepG2 and SMMC7721 cells transfected with control siRNA or HDAC6 siRNA, treated with the indicated concentrations of carfilzomib or ixazomib and analyzed as in (a). HDAC6 KD reduced the sensitivity of HCC cells to carfilzomib and ixazomib, two other proteasome inhibitors. **e** Correlation between E6AP or HDAC6 expression and the IC_50_ of ACY-1215 or bortezomib in liver cancer cell lines. E6AP/HDAC6 expression and IC50 of ACY-1215 and bortezomib were determined by immunoblot and CCK8 assay, respectively. The correlation was examined by Pearson’s correlation test. **f** Cell viability analysis of MHCC-97H cells transfected with control siRNA, E6AP siRNA, HDAC6 siRNA or FHL1 siRNA and treated with ACY-1215 (0.5 μM), bortezomib (3 nM) or ACY-1215 (0.5 μM) plus bortezomib (3 nM) for 48 h. The knockdown effects were assessed by immunoblot. Data shown are mean ± SD of triplicate measurements that have been repeated 3 times with similar results (a-g). The P values were generated using two-way ANOVA. **P* < 0.05, ***P* < 0.01. **g** Effects of ACY-1215 (ACY) and bortezomib (Bor) on common targets of E6AP and HDAC6. Immunoblot analysis of MHCC-97H cells treated with the indicated concentrations of ACY-1215, bortezomib or ACY-1215 plus bortezomib for 24 h. **h** Regualtion of ACY-1215 and bortezomib on deacetylation of FHL1 and TRIM3. Co-IP analysis of MHCC-97H cells transfected with FLAG-tagged FHL1 or TRIM3 and treated with the indicated concentrations of ACY-1215, bortezomib or ACY-1215 plus bortezomib for 24 h.

Figure. S14.


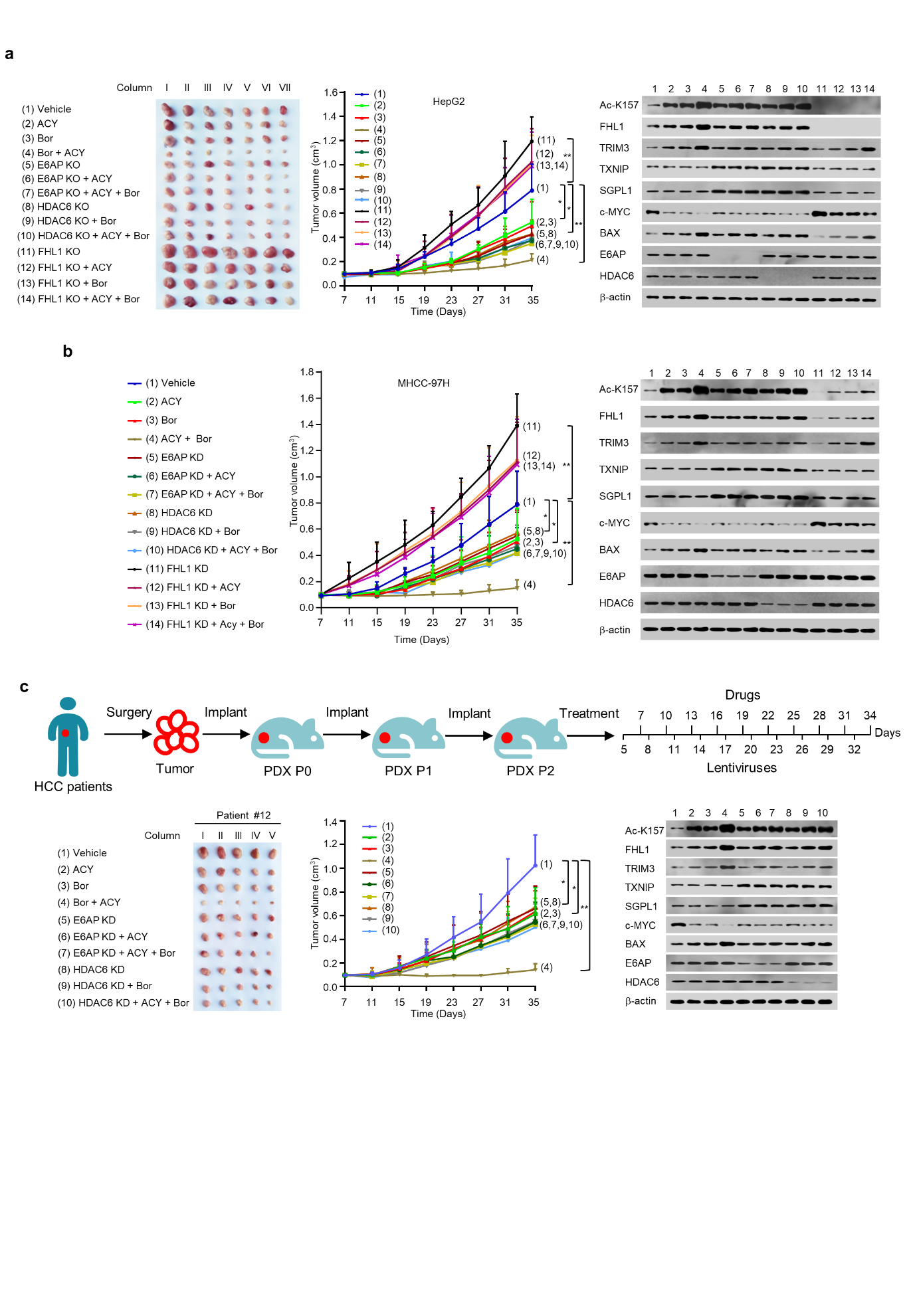


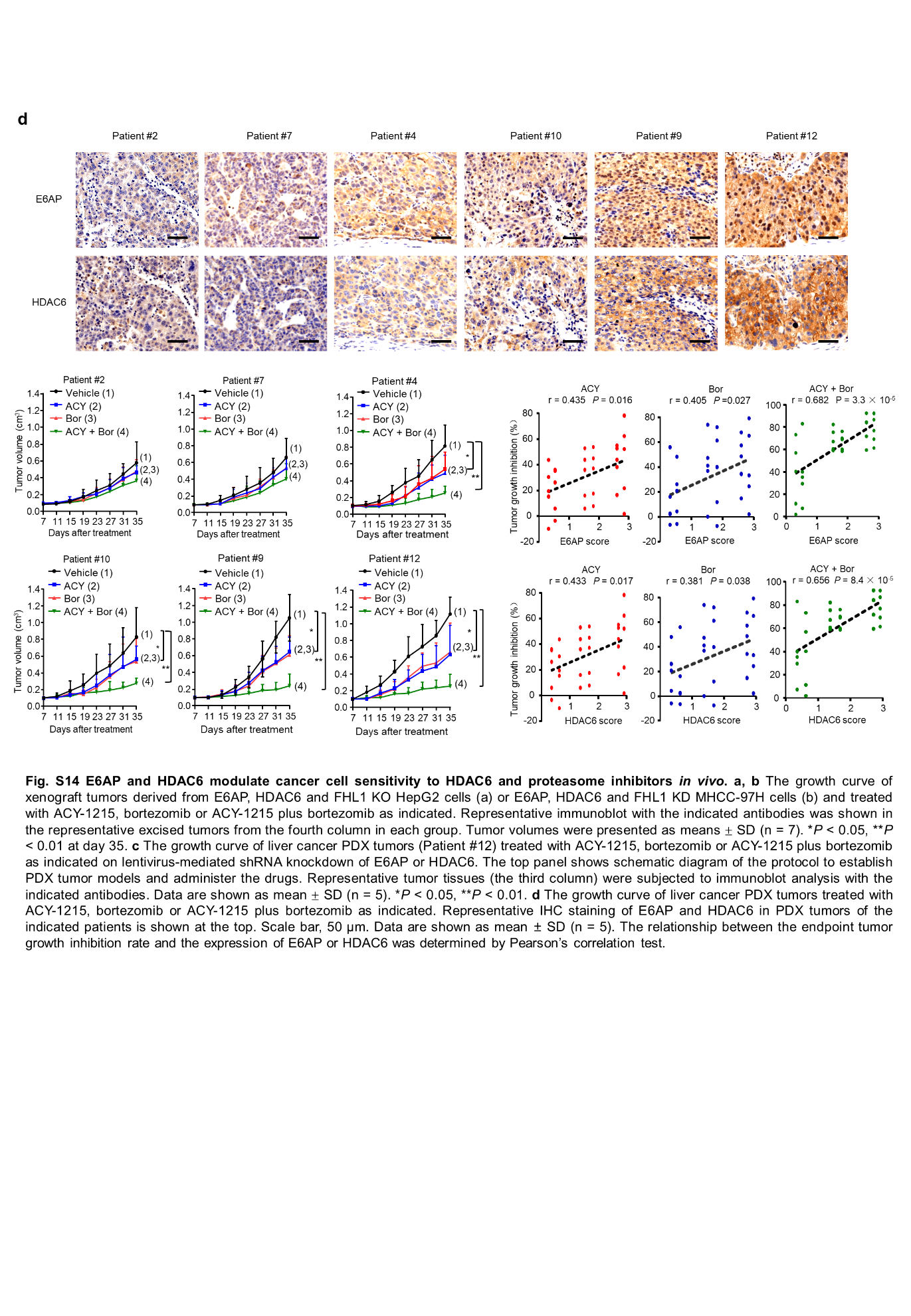


**Figure. S14 E6AP and HDAC6 modulate cancer cell sensitivity to HDAC6 and proteasome inhibitors *in vivo*. a, b** The growth curve of xenograft tumors derived from E6AP, HDAC6 and FHL1 KO HepG2 cells (a) or E6AP, HDAC6 and FHL1 KD MHCC-97H cells (b) and treated with ACY-1215, bortezomib or ACY-1215 plus bortezomib as indicated. Representative immunoblot with the indicated antibodies was shown in the representative excised tumors from the fourth column in each group. Tumor volumes were presented as means ± SD (n = 7). **P* < 0.05, ***P* < 0.01 at day 35. **c** The growth curve of liver cancer PDX tumors (Patient #12) treated with ACY-1215, bortezomib or ACY-1215 plus bortezomib as indicated on lentivirus-mediated shRNA knockdown of E6AP or HDAC6. The top panel shows schematic diagram of the protocol to establish PDX tumor models and administer the drugs. Representative tumor tissues (the third column) were subjected to immunoblot analysis with the indicated antibodies. Data are shown as mean ± SD (n = 5). **P* < 0.05, ***P* < 0.01. **d** The growth curve of liver cancer PDX tumors treated with ACY-1215, bortezomib or ACY-1215 plus bortezomib as indicated. Representative IHC staining of E6AP and HDAC6 in PDX tumors of the indicated patients is shown at the top. Scale bar, 50 μm. Data are shown as mean ± SD (n = 5). The relationship between the endpoint tumor growth inhibition rate and the expression of E6AP or HDAC6 was determined by Pearson’s correlation test.

Table S1. FHL1-interacting proteins identified by the yeast two-hybrid system

| **Abbreviation** | **Full name** |
| --- | --- |
| RIP140 | Receptor interacting protein of 140 kDa* |
| CHK2 | Checkpoint kinase 2* |
| CDC25C | Cell division cycle 25C* |
| 14-3-3ε | 14-3-3ε* |
| E6AP | E6-associated protein |
| HDAC6 | Histone deacetylase 6 |
| GAPDH | Glyceraldehyde 3-phosphate dehydrogenase |
| ENO1 | Enolase 1 |
| α-tubulin | α-tubulin |
| β-tubulin | β-tubulin |
| PAX7 | Paired Box Gene 7 |
| Tax1BP1 | Tax1-binding protein 1 |
| MSH2 | MutS protein homolog 2 |
| TCP1 | T-complex protein 1 |
| ZNF16 | Zinc finger protein 16 |
| ZFP95 | Zinc finger protein 95 |
| XPA | Xeroderma pigmentosum group A |
| tau | The microtubule-associated protein tau |
| DTX3 | Deltex 3 |

*Validated proteins in this laboratory.

**Table S2. E6AP-interacting proteins identified by the yeast two-hybrid system**

| **Abbreviation** | **Full name** |
| --- | --- |
| TRIM3 | Tripartite motif-containing 3 |
| TXNIP | Thioredoxin interacting protein |
| ERα | Estrogen receptor α* |
| TCAF1 | TRP channel-associated factor 1 |
| ANXA8 | Annexin A8 |
| EIF1 | Eukaryotic translation initiation factor 1 |
| PNKP | Polynucleotide kinase/phosphatases |
| CCDC9 | Coiled-coil domain containing 9 |
| HERC1 | HECT Domain and RCC1-like Domain 1 |
| PHKG2 | Phosphorylase kinase catalytic subunit gamma 2 |
| CEBPβ | CCAAT/enhancer-binding protein β |
| TRIP4 | Thyroid hormone receptor interactor 4 |
| TCS2 | Tuberous sclerosis complex 2* |

*The E6AP-interacting proteins reported in the literature.

**Table S3. HDAC6-interacting proteins identified by the yeast two-hybrid system**

| **Abbreviation** | **Full name** |
| --- | --- |
| TRIM3 | Tripartite motif-containing 3 |
| TXNIP | Thioredoxin interacting protein |
| IIp45 | Invasion inhibitory protein 45* |
| WDFY1 | WD repeat and FYVE domain containing 1 |
| SRSF3 | Serine and arginine rich splicing factor 3 |
| FDXR | Ferredoxin reductase |
| FOXJ3 | Forkhead box J3 |
| SEPT6 | Septin 6 |
| CCDC6 | Coiled-coil domain containing 6 |
| EIF2D | Eukaryotic translation initiation factor 2D |
| SMOC1 | SPARC related modular calcium binding 1 |
| PIAS2 | Protein inhibitor of activated STAT 2 |
| DDX6 | DEAD-box helicase 6 |
| MUL1 | Mitochondrial E3 ubiquitin protein ligase 1 |
| IRF7 | Interferon regulatory factor 7 |
| RSRC1 | Arginine and serine rich coiled-coil 1 |

*The HDAC6-interacting protein reported in the literature.

**Table S4. Characteristics of the established PDX models for liver cancer**

| **Case**  **No.** | **Gender** | **Age** | **Cancer Subtype** | **Cancer Stage** | **Treatment History** | **AFP**  **(μg/L)** | **Hepatitis**  **virus** | **Tumor formation*** |
| --- | --- | --- | --- | --- | --- | --- | --- | --- |
| #1 | Male | 50 | HCC | T3N0M0 | No | 1464.3 | HBV | Yes |
| #2 | Male | 59 | HCC | T1N0M0 | No | 3532.8 | HBV | Yes |
| #3 | Male | 51 | HCC | T2N0M0 | No | 2314.6 | HBV | No |
| #4 | Male | 66 | HCC | T2N0M0 | No | 1859.5 | HBV | Yes |
| #5 | Male | 60 | HCC | T3N0M0 | No | 6856.7 | HBV | Yes |
| #6 | Female | 47 | HCC | T1N0M0 | NO | 1352.9 | HBV | Yes |
| #7 | Male | 62 | HCC | T1N0M0 | No | 215.7 | HBV | Yes |
| #8 | Male | 38 | HCC | T1N0M0 | No | 2.4 | No | No |
| #9 | Male | 65 | HCC | T3N0M0 | No | 23289.4 | HBV | Yes |
| #10 | Male | 56 | HCC | T2N0M0 | No | 62.3 | HBV | Yes |
| #11 | Female | 62 | HCC | T2N0M0 | No | 2110.5 | HBV | No |
| #12 | Male | 63 | HCC | T3N0M0 | No | 11259.2 | HBV | Yes |
| #13 | Male | 40 | HCC | T1N0M0 | No | 13.3 | HBV | No |
| #14 | Male | 54 | HCC | T2N0M0 | No | 1689.67 | HBV | Yes |
| #15 | Female | 46 | HCC | T1N0M0 | No | 1010.54 | No | No |

*Fresh HCC tissues from each patient were transplanted into NOD/SCID mice and tumor formation was observed.

**Table S5. The cDNA target sequences of shRNA and siRNA**

| **Gene** | **Target sequence (5’-3’)** |
| --- | --- |
| E6AP | GAAGCAGTTGTATGTGGAA |
| UBE3B | TGTGGTATGTGTCCCTGGCT |
| UBE3C | TTACTTACA ACTCCTGTCCG |
| HDAC6 | CTGCAAGGGATGGATCTGAAC |
| HDAC10 | CAGGTGAACAGTGGTATAGCA |
| FHL1 | AAGGAGGTGCACTATAAGAAC |
| TRIM3 | CCACAAGAATGGCACATAT |
| TXNIP | CATCCTTCGAGTTGAATAT |
| SGPL1 | CCAGAGAGTTTATGGTCAA |
